# Development and assessment of immediate-release tablets containing clopidogrel bisulphate & aspirin—strategy for optimizing the combination formulation

**DOI:** 10.1371/journal.pone.0303705

**Published:** 2024-05-23

**Authors:** Shahnaz Usman, Muhammad Akram, Anab Usman, Sakina Fatima, Quamrul Islam

**Affiliations:** 1 Department of Pharmaceutics, RAK College of Pharmacy, RAK Medical and Health Sciences University, RAS, Al-Khaimah, UAE; 2 Department of Pharmaceutics, Faculty of Pharmacy, University of Karachi, Karachi, Pakistan; 3 Department of Medicine, Bedford Hospital National Health Services Trust, Bedford, United Kingdom; 4 Faculty of Pharmacy, Jinnah University for Women, Karachi, Pakistan; Kohat University of Science and Technology (KUST), PAKISTAN

## Abstract

The main goal of the study was to improve the compliance and convenience of patients by designing and development of an immediate release (IR) fixed-dose combination (Clopidogrel bisulphate and Aspirin) tablets. The proposed combination product utilizes Clopidogrel to protect the moisture-sensitive aspirin component, enhancing its stability against atmospheric conditions. Response-surface approach (Design Expert vs. 13) was used to generate this IR tablet by calculating the right composition of independent variables such as Microcrystalline cellulose 102, pregelatinized starch and Hydroxypropyl cellulose. 3^2^ factorial design was used to estimate the effects of these independent variables on the responses of dependent variables (disintegration & friability) and constructed a total of nine (9) formulations. Pre and Post formulation, quality control parameters were investigated as per pharmacopeia. A systematic approach was used for the optimization process and a prototype checkpoint batch (CPB) based on the better contrast of independent variables was prepared. In vitro analysis of formulations was carried out to estimate the responses. Friability was found in the range of 0.088–1.076%w/w, except F1 = 1.076 all are within limits (NMT 1.0%). Disintegration time was recorded 7.3 ± 1.20 as lower and 24.5 ± 1.63 min was the highest. The release of drugs from their dosage form was fast and rapid, for clopidogrel after 15min was 70.42–96.82% with SD ± 8.71 and aspirin was 69.88–91.49% in 15 min with SD ± 6.41, all the tablets were released more than 80% in 20 min. The stability outcomes of CPB tablets after 15 days of stress study (60 ± 2°C and 75 ± 5%) indicated good compatibility and stability of APIs with excipients. It was concluded that the direct compression method can be preferred to prepare a combination product with cost-effectiveness. It was also concluded that the proposed methodology could increase Aspirin’s stability and allow for an aqueous coating system to finish the product with a film coating. By using Design Expert software, the best composition of the formulation can be selected and optimized in a short period of time with minimum trial and errors. The results also demonstrated that the use of a fixed-dose combination tablet instead of the individual is expected to be more convenient to patients and thus improves patient compliance and decreases the occurrence of adverse effects and side effects.

## Introduction

Acute coronary syndromes (ACSs) are mostly initiated and developed due to platelet aggregation and thrombus formation. As per current guidelines, aspirin is recommended for the prevention of colorectal cancer along with its use in the prevention of cardiovascular diseases [1]. Its antiplatelet, anti-inflammatory, [2, 3] and proapoptotic effects [4] are almost similar to that of other antiplatelet drugs [2]. One of the good examples of such an antiplatelet drug is clopidogrel which is an irretrievable prodrug of similar structure (thienopyridine) that inhibits the P2Y12 of adenosine diphosphate receptors which play a vital role in platelet activation [5].

Hydroxy methyl glutaryl coenzyme A (HMG-CoA) reductase, involved in the production of cholesterol, is the rate-controlling enzyme of the mevalonate pathway. HMG-CoA Reductase accelerates the transformation of HMG-CoA to mevalonic acid which helps to decrease the plasma cholesterol levels and subsequently reduce the risk of cardiovascular disease (CVD). In these situations, antiplatelet therapy and antithrombotic therapy play a pivotal role to amend clinical outcomes and assist in lowering the incidence of crucial cardiac events [6–8].

Aspirin is a non-steroidal anti-inflammatory drug (NSAID) that works as an anti-inflammatory, analgesic and antipyretic agent but the frequent and extended use of aspirin in higher doses can cause interstitial nephritis, which can result in chronic kidney disease. Clopidogrel bisulfate is an antithrombotic agent. The combination of aspirin and clopidogrel is available in the market as a tablet dosage form such as film-coated, combination of film and enteric coating and bilayer tablets, and is the drug of choice for treatment of heart diseases. The chief purpose of using dose combination in a tablet is to substitute for two separate tablets of clopidogrel and aspirin, making it more convenient and easier for patients to consume, hence improving the chances of compliance. In case of polypharmacy, the combination formulation also helps to reduce the risk of adverse and side effects.

In previous studies, researchers have endeavored to formulate a bilayer floating tablet using natural gums, observing a sustained effect from the combination [9]. In another study, solid dispersions were prepared utilizing a similar combination, allowing for the simultaneous determination of acetylsalicylic acid and clopidogrel bisulfate, and subsequent release from the solid dispersions [10]. Many marketed tablets are either bilayered or film-coated, releasing both drugs simultaneously, which could potentially decrease the bioavailability of clopidogrel [11–13], albeit without compromising its efficacy. However, certain literature suggests a synergistic effect [14–17], which could lead to adverse effects such as unusual bleeding or severe abdominal pain.

The main objective of the present study was to design and formulate an immediate-release tablet (IRT) of Clopidogrel bisulphate and Aspirin of strength 75/75 mg in one formulation without making the bilayer of APIs or using coating techniques. The efforts were made to improve the dissolution of clopidogrel bisulphate and aspirin by using different approaches, as both drugs are categorized as BCS class II agents. In the proposed combination product, the aspirin component is protected by the Clopidogrel compound to shield it from atmospheric conditions, such as changes in relative humidity during coating, storage, and distribution. Aspirin is a highly moisture-sensitive drug that is easily hydrolyzed. Hence, the proposed methodology is aimed at increasing the stability of Aspirin and allow for the application of an aqueous coating system to finish the product with a film coating. The study also focused on evaluating and optimizing the process parameters, especially the selection of disintegrants that must be compatible with APIs as well as to make a tablet an immediate-release formulation that should have a potential to release clopidogrel and aspirin quickly when exposed to gastrointestinal milieu and hence contributing in reduction of the errors of multi-drug administration. Finally, the stability of formulated tablets was assessed by a validated HPLC analytical method [18] for their quantitative estimation before and after.

## Material and methods

### Materials

Clopidogrel bisulphate (CB) was obtained as a gift sample from Aurobindo Pharma, Hyderabad, India. Aspirin (A) was also acquired as a gift sample from the Faculty of Pharmacy, Karachi University, Pakistan, the source of material was JQC (Huayin) Pharmaceutical Co., Ltd, China. Microcrystalline cellulose 102 (MCC), pregelatinized starch, mannitol, stearic acid, hydroxypropyl cellulose, polyethylene glycol 6000, hydrogenated castor oil, colloidal silicon dioxide, octane-1-sulfonic acid sodium salt (Fisher Scientific), orthophosphoric acid (Merck), acetonitrile (Merck), double distill water HPLC grade (Merck) and distill water was freshly prepared by distillation method.

### Methods

#### Ethical statement

The approval of the Research and Ethics Committee of RAKMHSU was obtained before the work was initiated. REC Approval # RAKMHSU-REC-256-2020-F-P.

#### Experimental design

The most important step of formulation development is the selection of appropriate excipients with correct compositions to generate a design of dosage form to match the desired requirements and properties precisely [19–24]. The pre-formulation studies were carried out to find out the impact of excipients on the basic properties and characteristics of other excipients and on the APIs. The quantitative data of the preformulation study provided information regarding the interpretation of interactions among the excipients and with API.

#### Drug-drug compatibility study

In acute coronary syndromes, aspirin (A) and clopidogrel (CB) are essential components of medical therapy, especially for patients undergoing coronary artery stenting as well as for the prevention of ischemic stroke [25].

The most critical problem that needs to be addressed during the development of combination formulation, especially when the APIs are aspirin and clopidogrel, is the stability of both compounds in one product. To evaluate the compatibility and level of degradation of aspirin in the presence of clopidogrel, a stability study was carried out at an elevated temperature (50±2 °C). An equal quantity of aspirin and clopidogrel were mixed and kept in a hot air oven for 3 weeks. It was analyzed periodically after 0, 1, 2 and 3 weeks (S2 Fig).

#### Selection of excipients and their measurements

For the construction of acceptable tablets, DSC and FTIR spectroscopy was performed for pure drug and the blended mixture of drug and excipients, as well as the bulk and tapped density, angle of repose, compressibility index and Hausner ratio for different excipients were carried out individually and in combined mixture blends to study the impact of their essential and fundamental properties on the pharmaceutical structure of dosage form.

#### Preparation of tablets by direct compression

After scrutinizing the fundamental characteristics of all the ingredients, 3^2^ factorial designs for two different API tablet formulations were used for their construction separately, total (9) nine formulations for each API using Design Expert (DE): version 13.0.13, were prepared. The first oral tablet was prepared for aspirin where Microcrystalline cellulose 102 (MCC) and pregelatinized starch were selected as independent variables and the second was clopidogrel bisulphate formulation where Microcrystalline cellulose 102 and Hydroxypropyl cellulose (HPC) were used with their three different concentrations i.e. low, medium, and high to prepare different batches and their responses were assessed.

All the ingredients were weighed carefully and dry-mixed to get a homogenous mixture of powder. The powder was passed through mesh # 40 to get the uniformity of particle size and distribution to achieve the new formulation (combined formulation of A+ CB) well within the proposed limit of desired characteristics. Aspirin tablets were compressed with 7.00 mm round shape plain punches at a theoretical weight of 120 mg ± 7.5% whereas CB was compressed with 11.00 mm (aspirin tablet inside) at a theoretical weight of 400 mg.

#### Formulation optimization

The first sample tablets were prepared after the screening of excipients which may cause the excipient-excipients and drug-excipients interaction if the selection of these were not done properly. The basic information of tablets was gathered from the marketed brands of the same drugs combination.

This study was carried out to develop a combined formulation of an immediate-release tablet consisting of clopidogrel bisulphate and aspirin in a concentration of 75mg /75 mg. The preliminary dissolution study of the formulated tablets was carried out and the release pattern of drugs from the combined formulation (A+CB) was calculated which confirmed the suitability of excipients. To adjust the desired shape of release, the series of formulations (9) were constructed with identical ingredients with the help of software Design- Expert 13.0.13, State Ease Inc.

In the proposed study, two-factor three-level factorial designs were applied in the formulation development of aspirin tablets. One factor was MCC pH 102 which was used at three levels 10% (12 mg/tab), 15% (18 mg/tab) and 20% (24 mg/tab). The second factor was pregelatinized starch which was used at 5% (6 mg/tab), 7.5% (9 mg/tab) and 10% (12 mg/tab).

The same factorial design was used for the construction of clopidogrel tablets. One factor was MCC pH 102 which was used at three levels 10% (40 mg/tab), 20% (80 mg/tab) and 30% (120 mg/tab) Whereas the second factor was hydroxypropyl cellulose (HPC) low-substituted which was used at 4% (16 mg/tab), 8% (32 mg/tab) and 12% (48 mg/tab). The tablets were prepared by dry blending and compression. First, the aspirin tablets were prepared and evaluated for pharmaceutical quality attributes. Secondly, the clopidogrel tablets were prepared by keeping the aspirin tablets inside the clopidogrel and the release of A and CB from the combined product was analyzed.

#### In-vitro evaluation of optimized batches

To assess their performance, all nine (9) formulations were examined for dissolution and other quality control tools of tablets. The tablets were analyzed for their elegancy with adequate consistency of weight, size, hardness, disintegration and friability.

#### Checkpoint batch (CPB)

Two responses of independent factors, disintegration and friability were designated for further adjustment and finalization of the best one batch among the optimized batches. Finally, aspirin (A8) having microcrystalline cellulose pH 102 (18mg) and pregelatinized starch (12mg) and clopidogrel (F8) with MCC pH 102 (80 mg) and hydroxypropyl cellulose (48 mg) were selected as the optimized batch (CPB) with the target of less than 0.5% friability and less than 15 minutes disintegration time. A Check Point Batch (CPB) was prepared and evaluated as per the tests described to confirm the reliability and validity of the optimization design.

#### Stability studies

The CPB tablets were kept for stress testing at 60 ± 2°C and 75 ± 5% R.H as per ICH guidelines [26, 27]. This Stress study is useful in establishing the degradation pathways that can help to predict and establish the packaging condition of the product. The tablets were placed into amber bottles, studies were done for 15 days and were evaluated at three intervals i.e. initially (zero-day), after one week (7 days) and 2^nd^ weeks (15 days).

## Results and discussion

**Fig 1:**
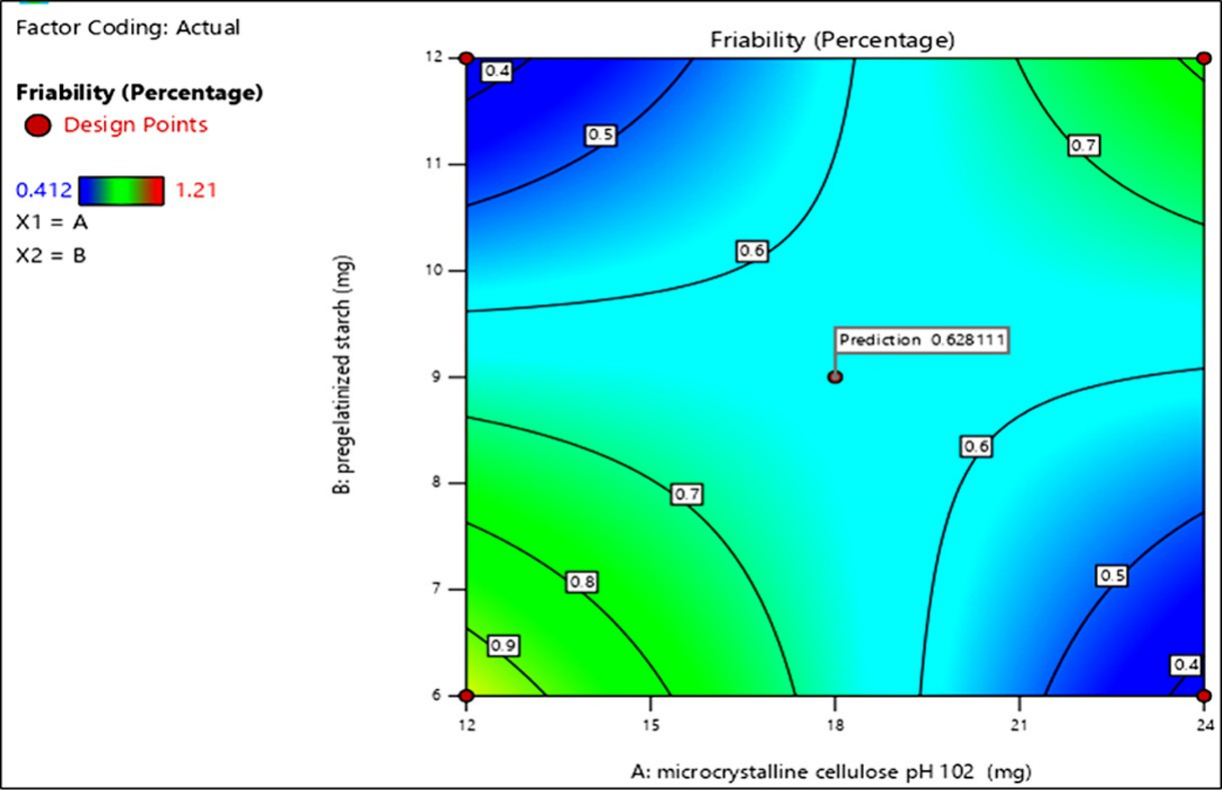
Contour plot of standard error of design of optimization batches (% Friability). Optimum predicted response value for friability (in percent) when the change in two variables (MCC pH 102 and pregelatinized starch) occurred.

**Fig 2:**
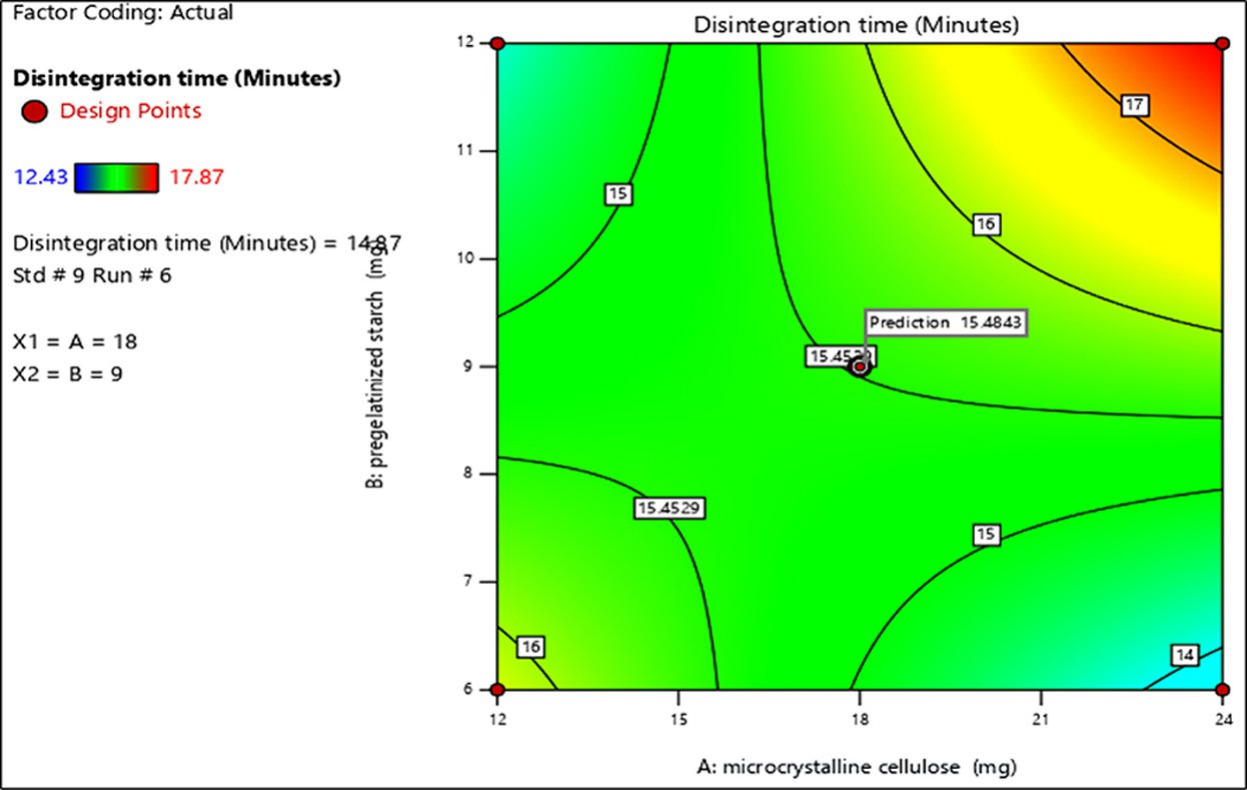
Contour plot of standard error of design of optimization batches (Disintegration time). Predicted response value of disintegration time (in minutes) upon alteration in two variables (MCC pH 102 and pregelatinized starch).

Initially, the tablets were formulated by using the standard characteristics of different excipients and evaluated for disintegration and friability. The obtained values were put into the Design Expert (Version 13). The formulation with microcrystalline cellulose pH 102 and pregelatinized starch was selected for Aspirin tablets; MCC pH 102 and hydroxypropyl cellulose were selected for clopidogrel tablets respectively, the same excipients were used as variables for the optimization of prototype formulation studies (Figs 1 and 2).

The Contour Plot of Standard Error gives ideas to manage the expectations of the formulation design. The plot was used to evaluate the response values when the change in 2 variables occurred [28–30]. The combination of independent variables such as X1 = microcrystalline cellulose pH 102 and X2 = pregelatinized starch was used to estimate the impact of responses on the disintegration time and % friability, used as dependent variables. In this respect, color-coded regions help to investigate the error of responses. The low responses are indicated by a cool blue color whereas high responses are in yellow (Figs 1 and 2).

**Fig 3:**
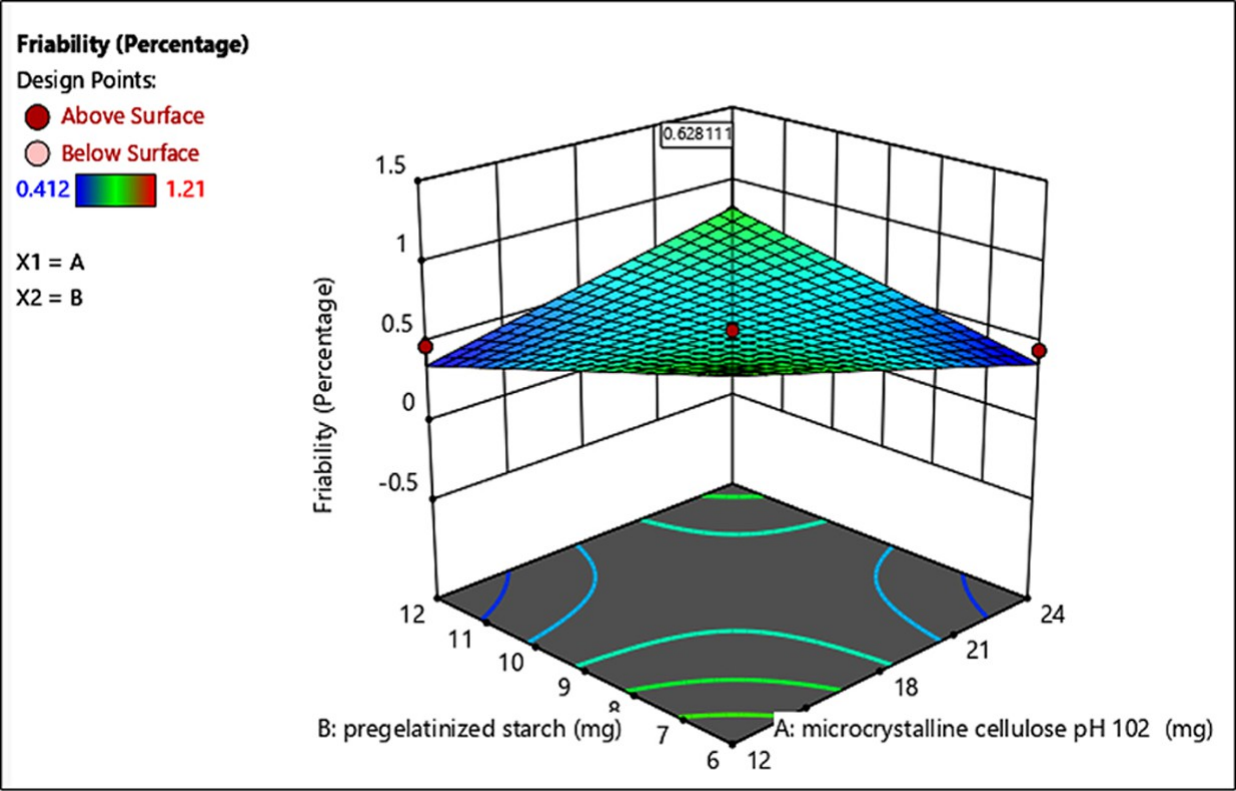
3D surface plot of standard error of design of optimization batches (% Friability). 3D surface plot visualization of friability responses as a function of variables (MCC pH 102 and pregelatinized starch).

**Fig 4:**
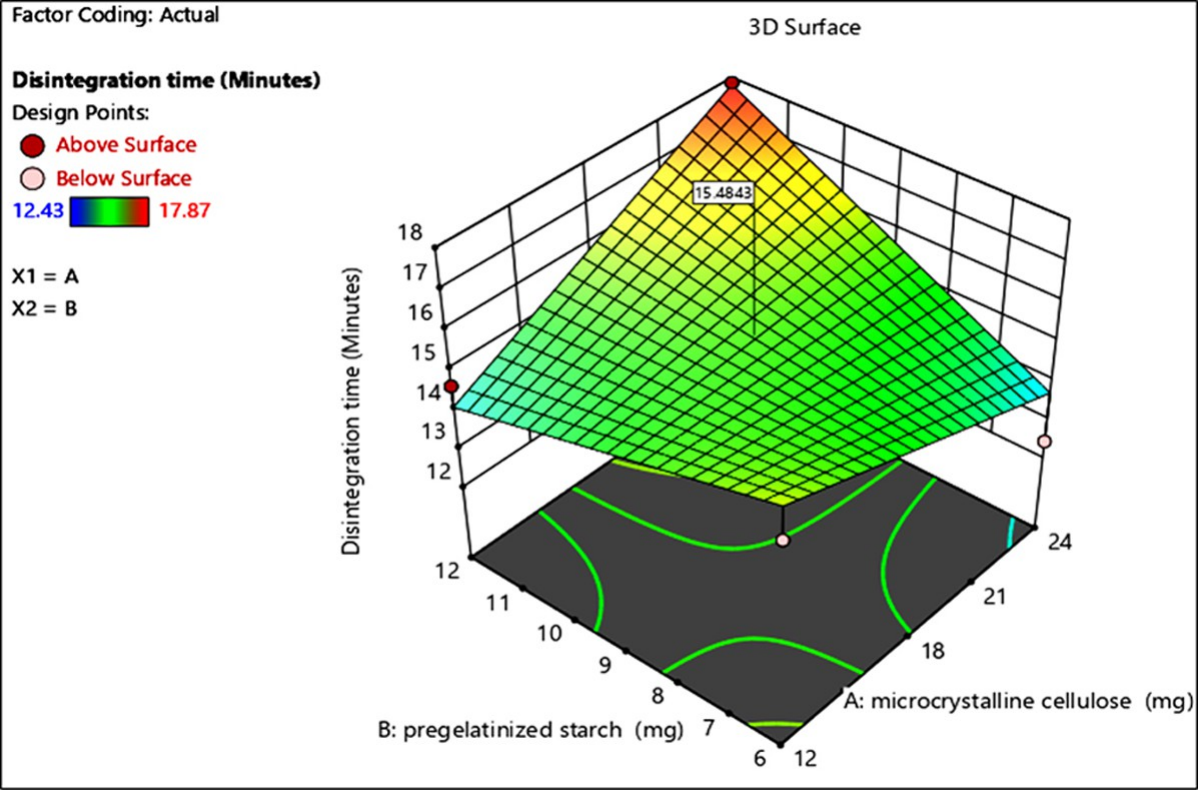
3D surface plot of standard error of design of optimization batches (Disintegration time). Graphical visualization of disintegration time responses as a function of variables via a 3D surface plot.

Whereas the 3D plot provides a comprehensive visualization of how these variables interact to affect the standard error, showing peaks and valleys in the landscape of optimization possibilities. This visualization helps in identifying optimal conditions for minimizing variability in friability and disintegration during batch production (Figs 3 and 4).

A response surface model with a central composite design was selected for this experiment. Upon satisfactory results of friability and disintegration, the prototype formulation with MCC pH 102, pregelatinized starch and hydroxypropyl cellulose was selected for further optimization.

Table 1 shows the results of the compatibility study of Aspirin with Clopidogrel bisulphate. The combination of these two APIs is very frequently used to prevent heart attacks and blockage of stents. The literature survey revealed that a combination may provide better secondary prevention of stroke than one antiplatelet alone. The focus of the present study was to develop a prototype formulation with their optimized characteristics. In this regard, the first highlighted step is to estimate the level of compatibility of these two APIs with each other (Table 1).

**Table 1:** Drug-drug compatibility study of aspirin & clopidogrel bisulphate.

Sample	0 week		1st week		2^nd^ week		3^rd^ week	
	A	CB	A	CB	A	CB	A	CB
**Sample-1** 60/80 μg/ml	2109636	2185357	2191537	2227075	2182482	2211101	2089607	2243089
	2118040	2190546	2174443	2217229	2166354	2195433	2110041	2261417
**Mean**	2113838	2187952	2182990	2222152	2174418	2203267	2099824	2252253
**% CV**	**0.2811**	**0.1677**	**0.5537**	**0.3133**	**0.5245**	**0.5028**	**0.6881**	**0.5754**
**Sample-2** 60/80 μg/ml	2123419	2167288	2093854	2175591	2145460	2218576	2137829	2261887
	2144250	2191470	2111980	2188325	2142984	2215636	2160681	2286404
**Mean**	2133835	2179379	2102917	2181958	2144222	2217106	2149255	2274146
**% CV**	**0.6903**	**0.7846**	**0.6095**	**0.4127**	**0.0817**	**0.0938**	**0.7518**	**0.7623**

CV = Percentage coefficient of variation

μg/ml = microgram per milliliter

**Fig 5:**
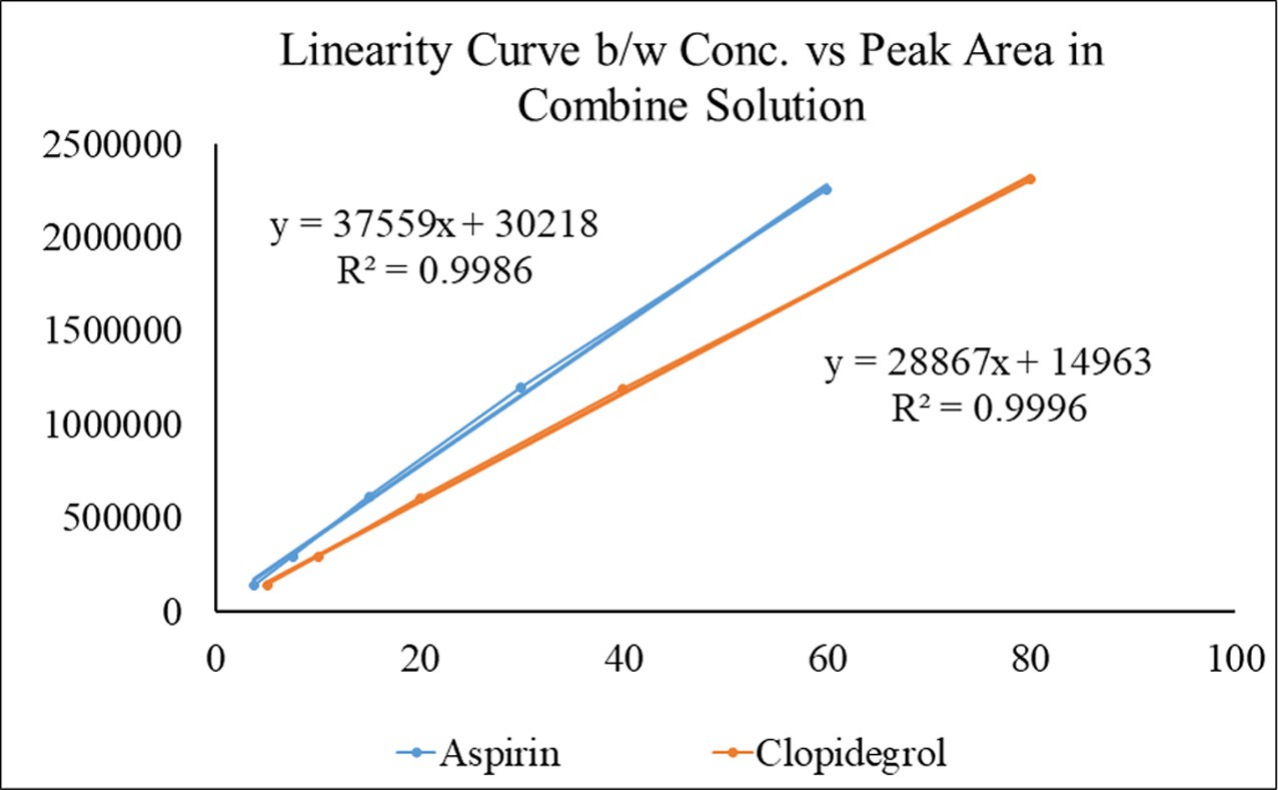
Linearity curve between concentration versus peak area in a combined solution. The calibration curve shows linearity over the concentration range of 60–0.117μg/ml for aspirin and 80–0.156 μg/ml for clopidogrel.

**Fig 6:**
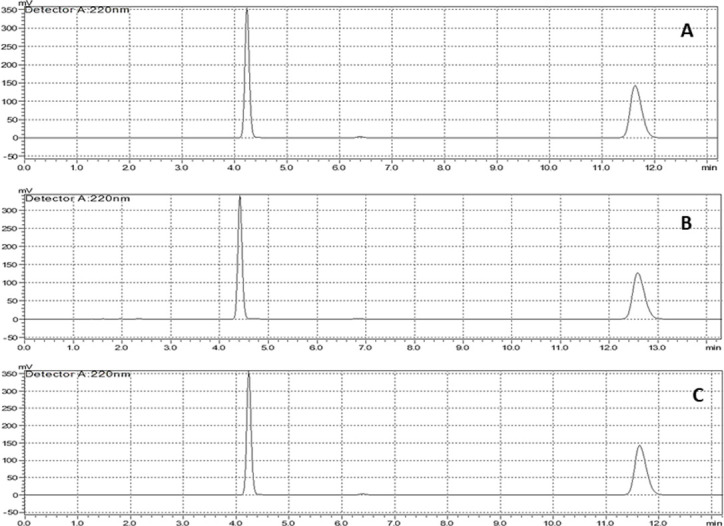
Chromatograms of drug-drug compatibility (60/80 μg/ml). A) Day 1^st^; B) Day 7^th^; C) Day 21^st^ sample.

The HPLC analytical method was first developed and validated (Fig 5) (S3 Fig) [18] then was used to calculate the interaction or decomposition of aspirin and clopidogrel mixture at elevated temperature (50±2 °C) for 3 weeks. After 3 weeks of analyses, it was found that the recovery of the peak area of two samples were 99.34 and 100.72% for aspirin and 102.94 and 104.35% for clopidogrel with respect to zero-week (day one) peak area (Table 1) (Fig 6). Whereas the %CV of aspirin for Sample_1A_ = 0.6881 and Sample_2A_ = 0.7518; Sample_1CB_ = 0.5754 and Sample_2CB_ = 0.7623 for clopidogrel respectively between two peak area of each sample which indicated the precision and affinity of two drugs. The assessment of drug-drug compatibility provided an important prerequisite before actual formulation to confirm that the drugs do not affect the shelf stability of product and stipulate the idea of an acceptable, safe and efficacious formulation.

**Fig 7:**
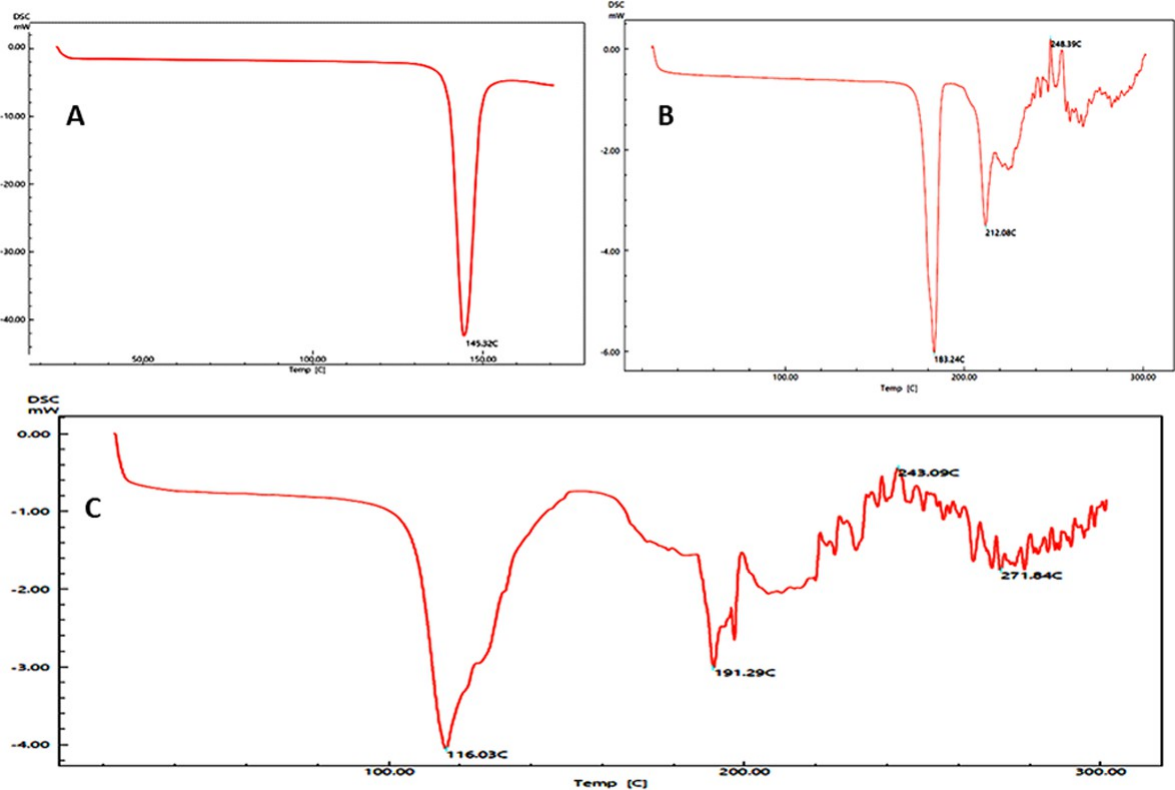
Differential Scanning Calorimetry (DSC) study of aspirin and clopidogrel. a) Aspirin; b) Clopidogrel bisulphate; c) Physical mixture of aspirin & clopidogrel bisulphate.

Construction of a new formulation or the modification of an existing one required a preformulation study of excipients and APIs to select the most appropriate combinations for the desired dosage form [31–33]. In this regard, the assessment of physical, chemical, and pharmaceutical characteristics related to molecules play a vital role and provides sufficient knowledge about the performance of a substance. The DSC of the aspirin and clopidogrel was carried out separately and in a mixture to explain their thermal stability, type of crystallization and chemical interactions of these compounds (Fig 7). The untreated clopidogrel confirmed a specific endothermic peak at 183.24 °C which links with its melting point (176.8 °C), this study is supported by the works done in the past [34–36]; indicated that CB was in the polymorphic form I, has slightly higher temperatures as compared to the polymorphic form II, is in good agreement with the values published in the literature [37, 38]. On the other hand, aspirin showed a sharp endothermic peak at 145.32 °C which corresponds to the characteristic melting temperature of aspirin, providing valuable information about its thermal properties and purity. The sharp peak indicated the crystalline nature and purity of the aspirin. The obtained thermogram of aspirin is almost the same as that of the previous studies [39–41]. The thermogram of the physical mixture of clopidogrel and aspirin illustrated that there is no interaction between these two APIs but the shifting of peaks with phase transitional energy took place. Two unique peaks appeared at 191.29 °C for clopidogrel and at 116.03 °C for aspirin respectively which were different from the peaks of the individual components before mixing.

**Fig 8:**
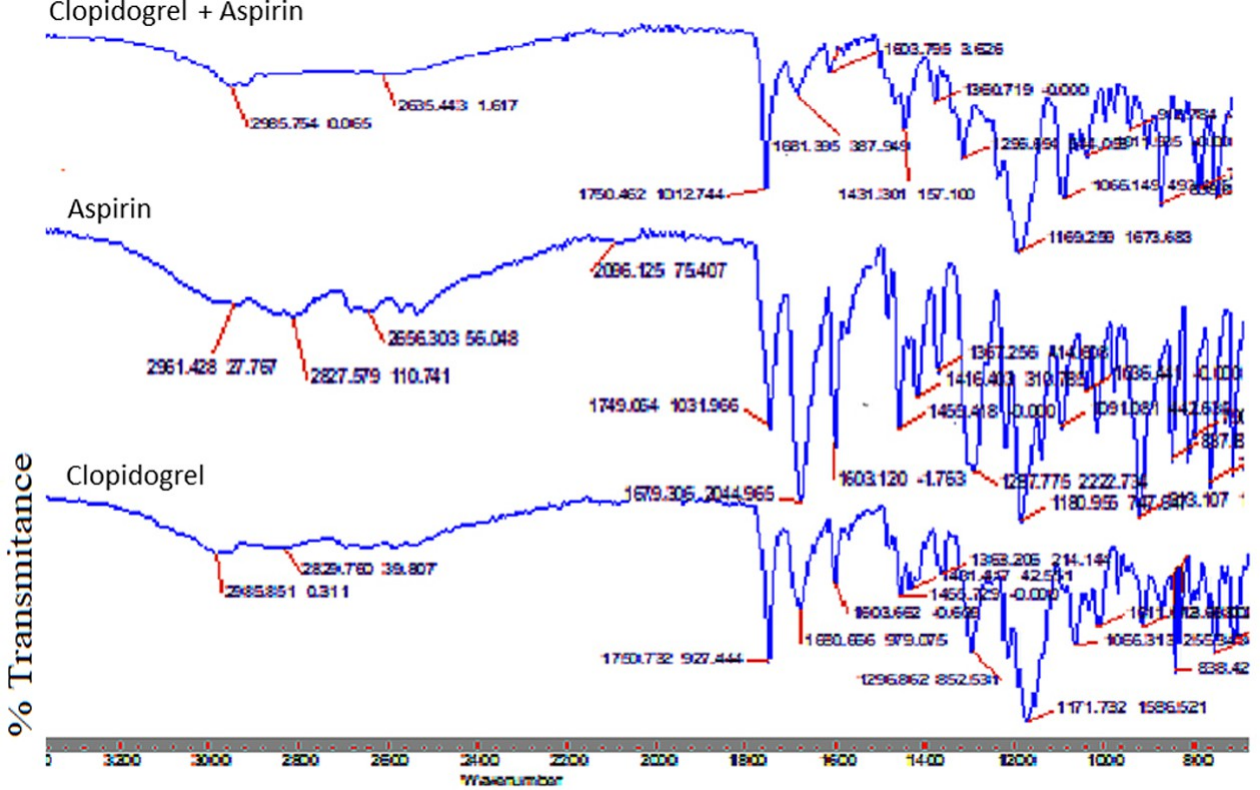
FTIR spectra of clopidogrel, aspirin and their physical mixture. Clopidogrel ‐ Characterize the unique absorption bands; Aspirin- indicates principal functional group; Clopidogrel + Aspirin ‐ Physical mixture is to characterize their molecular compositions and interactions assessment.

The compatibility of aspirin and clopidogrel was also analysed by FTIR by comparing their functional groups individually and in their mixture (Fig 8). In clopidogrel bisulphate (pure), a strong absorbance band was found at 1750.732 cm^–1^ due to C = O stretching vibrations, at 1171.732 due to C-O stretching and at 2986–2830 cm^–1^ due to O–H stretching of the hydrogen sulfate moiety. This study aligns well with the values published in the literature [37, 38, 42]. In case of aspirin, carboxylic acid is the principal functional group. The IR spectra of aspirin demonstrated the stretching vibration of C = O bonds between the 1679.306–1749.064 cm^-1^ and a band at 1603.12 cm^-1^ is accredited with the vibration of the benzene ring whereas multiple bands are in the range of 1600–1000 cm^-1^ [43]. In the physical mixture of aspirin and clopidogrel (Fig 8) there are no major transformations in the prominent peaks of both the drugs i.e clopidogrel and aspirin [42].

**Fig 9:**
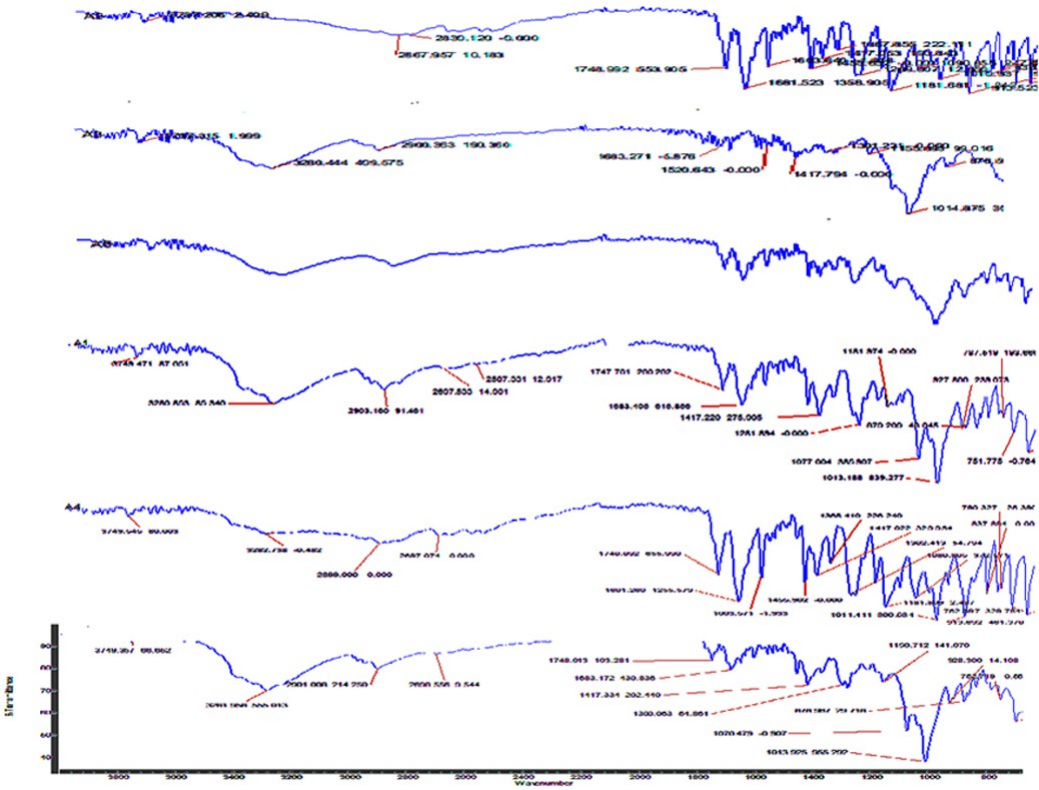
FTIR spectra of aspirin formulations.

**Fig 10:**
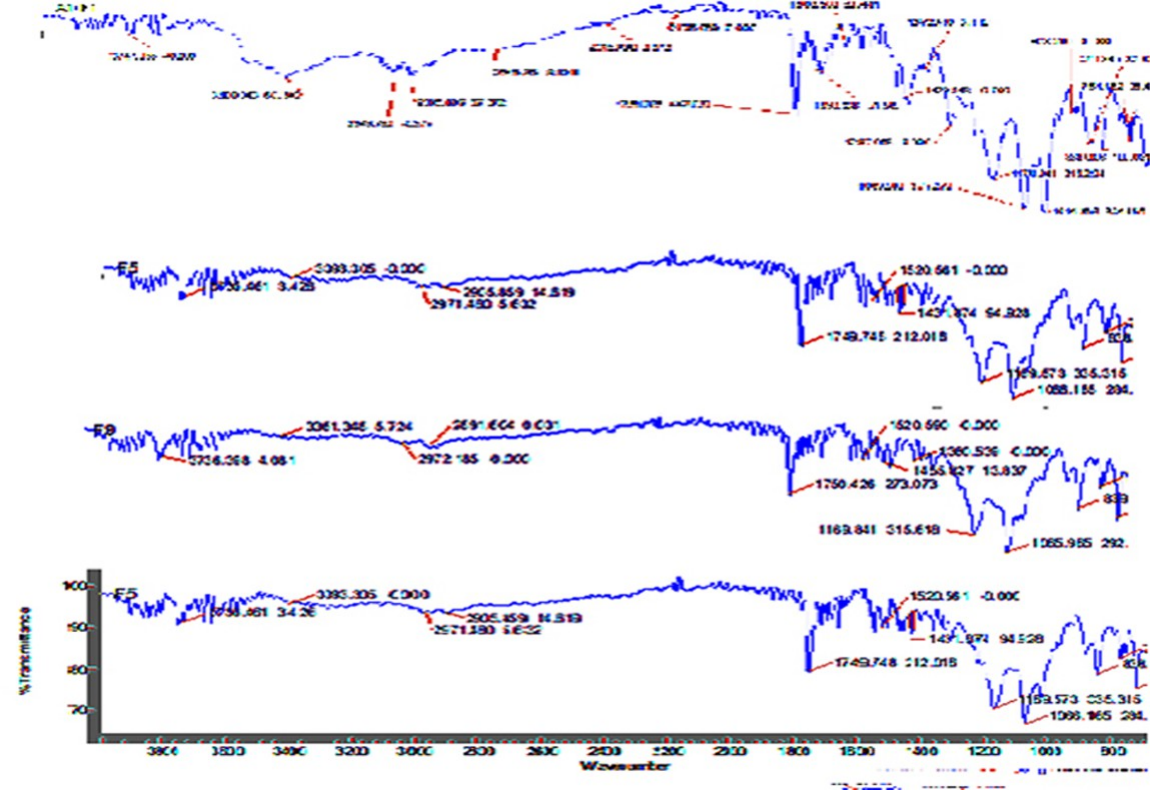
FTIR spectra of clopidogrel & aspirin combination formulations.

The compatibility of aspirin and clopidogrel with different excipients was checked by confirming the functional groups (shifting) in their prepared formulations blends. (Figs 9 and 10). The IR spectra suggested that there were no significant changes in the peaks, thus, it is concluded that the excipients used in the formulations are very much compatible with both the drugs. This study provides a good understanding of the existing literature [40, 42, 43].

The pre-formulation studies for the drug and excipients were also done by using different parameters like bulk density (BD) tapped density (TD), compressibility index (Carr’s index), Hauser ratio (H) and angle of repose separately (Table 2). The same parameters were used to analyse the flow properties of prototype aspirin powder blends (Table 3) and clopidogrel bisulphate powder blends (Table 4). These characteristics helped to determine the best excipients to be used in the formulation development of IRT. Hydroxypropyl cellulose (HPC) works as a viscosity-enhancing agent that develop high plastic deformation and good compatibility in the formulation. Pregelatinized starch makes better flowability during the tableting process. Whereas the PEG 6000 contributes to the proper mixing of powder components into “slugs” which helps to recompress the tablets in a good and satisfactory way [44–50].

**Table 2:** Preformulation studies of active and excipients.

Material	Bulk Density (g/ml)	Tapped Density (g/ml)	Carr’s Index (%)	Hauser’s Ratio	Angle of Repose* (θ)
Clopidogrel Bisulphate	0.545	0.654	16.602	1.199	19.800
Aspirin	0.714	0.769	7.152	1.077	44.035
Hydroxypropyl cellulose	0.370	0.476	22.270	1.286	36.240
Pregelatinize starch	0.633	0.791	19.975	1.249	38°
Microcrystalline cellulose	0.34	0.490	30.140	1.44	36 ± 0.014
Mannitol	0.476	0.667	28.636	1.4	33.99±0.011
Stearic acid	0.580	0.676	14.201	1.166	
Polyethylene glycol 6000	0.625	0.7143	12.502	1.143	38.589
Colloidal silicon dioxide	0.047	0.058	19.050	1.235	‐‐‐‐‐

*Angle of Repose = 16–31 (Good to Excellent)

*Flow properties of powder blends based on Mean ± SD; N = 3

1. % Carr’s index = ≤ 22 (Passable to Fair)

2. Hausner’s Ratio = ≤1.28 (Passable to Good

**Table 3:** Pre-formulation studies of prototype formulations of aspirin.

Formula Code	Bulk Density (g/ml)	Tapped Density (g/ml)	Flow Properties*		
			Carr’s Index %	Hausner’s Ratio	Angle of Repose* θ
**A1**	0.724 ± 0.008	0.80 ± 0.006	10.54	1.105	29.466±0.34
**A2**	0.723 ± 0.005	0.903 ± 0.006	24.98	1.249	24.513±0.36
**A3**	0.669 ± 0.007	0.84 ± 0.006	25.49	1.255	25.688±0.21
**A4**	0.711 ± 0.005	0.74 ± 0.005	3.46	1.035	26.660±0.41
**A5**	0.682 ± 0.008	0.86 ± 0.008	26.1	1.261	27.176±0.22
**A6**	0.707 ± 0.007	0.86 ± 0.005	21.5	1.215	32.822±0.51
**A7**	0.710 ± 0.005	0.736 ± 0.006	3.76	1.038	26.565±0.32
**A8**	0.714 ± 0.004	0.893 ± 0.005	25.04	1.25	31.467±0.31
**A9**	0.681 ± 0.006	0.859 ± 0.003	26.21	1.262	22.410±0.33

*Results based on (Powder Blends) Mean ± SD; N = 3

**Table 4:** Pre-formulation studies of prototype formulations of clopidogrel bisulphate.

Formula Code	Bulk Density (g/cc)	Tapped Density (g/cc)	Flow Properties*		
			Carr’s Index %	Hausner’s Ratio	Angle of Repose* θ
**F1**	0.398 ± 0.018	0.622 ± 0.016	56.281	1.563	43.950±0.31
**F2**	0.349 ± 0.055	0.668 ± 0.021	91.485	1.915	40.696±0.16
**F3**	0.337 ± 0.017	0.607 ± 0.023	80.012	1.800	37.104±0.21
**F4**	0.396 ± 0.025	0.659 ± 0.032	66.591	1.666	41.640±0.31
**F5**	0.355 ± 0.009	0.600 ± 0.018	69.155	1.692	35.004±0.23
**F6**	0.35 ± 0.027	0.574 ± 0.035	63.910	1.639	27.294±0.41
**F7**	0.402 ± 0.015	0.658 ± 0.021	63.731	1.637	39.83±0.32
**F8**	0.400 ± 0.044	0.667 ± 0.033	66.683	1.667	30.071±0.33
**F9**	0.390 ± 0.026	0.607 ± 0.033	55.547	1.555	38.45±0.35

*Results based on (Powder Blends) Mean ± SD; N = 3

After the preformulation investigation of powder blends, compression process was carried out by the direct compression method to get a prototype optimized formulation (Table 5). The evaluation of pharmaceutical parameters for all formulated tablets (F1-F9) was carried out to ensure that the devised product must fulfill the expectation of official compendia (USP-36) (Table 6). The appearance of all tablets was elegant, round and white in color. The weight of the tablets was adequately controlled, and consistency of weight was in the range of 514.043±2.18 to 528.536±1.25 mg/tab (Table 6). The friability was in the range of 0.088–1.076%w/w, all the tablets were found within the pharmacopeial acceptance limit (NMT 1.0%) except F1 = 1.076 which is little bit out of targeted value whereas disintegration time was recorded 7.3±1.20 as lower and 24.5±1.63 min was the highest (Table 6). It is supposed that the disintegration time of immediate-release tablets (IRT) should be within 2.5 to 10 min. but as an uncoated tablet, it can go up to 30 min.

**Table 5:** Design of prototype formulations for aspirin & clopidogrel.

CP Formulation, F8 (mg/ tablet)			
Materials (mg/tab)	F8	Materials (mg/tab)	A8
**Clopidogrel Bisulphate** (Equal to 75mg clopidogrel)	97.5	**Aspirin**	75
Microcrystalline cellulose 102	80	Microcrystalline cellulose 102	18
Hydroxypropyl cellulose	48	Pregelatinize starch	12
Mannitol	142.8	Mannitol	14
Polyethylene glycol 6000	20		
Hydrogenated Castor Oil	3.3	Stearic acid	1
Colloidal Silicon Dioxide	8		
**Total Weight**	**400**	**Total Weight**	**120**

**Table 6:** Analysis of pharmaceutical parameters of combined formulated tablets of A & CB.

Formula Code	Weight variation (mg)	Diameter (mm)	Hardness (Kg)	Friability % w/w	Disintegration time (Min)	Drug content (%)	
						A	CB
**F1**	519.493±3.35	11.1±0.85	5.25±1.66	1.076	9.72±2.81	102.41±1.67	101.5±1.33
**F2**	527.057±1.87	11.13±0.77	4.88±0.85	0.394	7.3±1.20	102.2±1.89	102.2±1.89
**F3**	525.936±2.42	11.1±0.90	5.38±0.95	0.176	21.41±2.35	104.6±2.06	99.37±1.36
**F4**	518.864±2.82	11.14±0.59	5.5±1.08	0.606	16.33±1.93	101.11±2.19	102.04±1.93
**F5**	527.114±1.92	11.16±0.45	5.0±0.91	0.321	17.21±1.06	102.33±1.79	102.68±2.09
**F6**	528.536±1.25	11.09±0.60	5.38±1.03	0.155	24.5±1.63	99.58±1.55	105.58±2.35
**F7**	514.043±2.18	11.16±0.39	5.25±1.5	0.423	23.75±2.78	101.35±1.19	98.35±1.09
**F8**	521.107±1.98	11.17±0.43	4.75±1.32	**0.177**	**12.03±1.98**	100.01±0.83	102.41±1.67
**F9**	525.742±2.18	11.15±0.42	4.88±0.85	0.088	12.675±2.91	102.50±0.69	102.50±1.69

**Weight variation =** based on average of 15 tablets ±SD

**Diameter (mm)** = based on average of 10 tablets ± SD

**Hardness (Kg)** = based on average of 5 tablets ± SD

**Fig 11:**
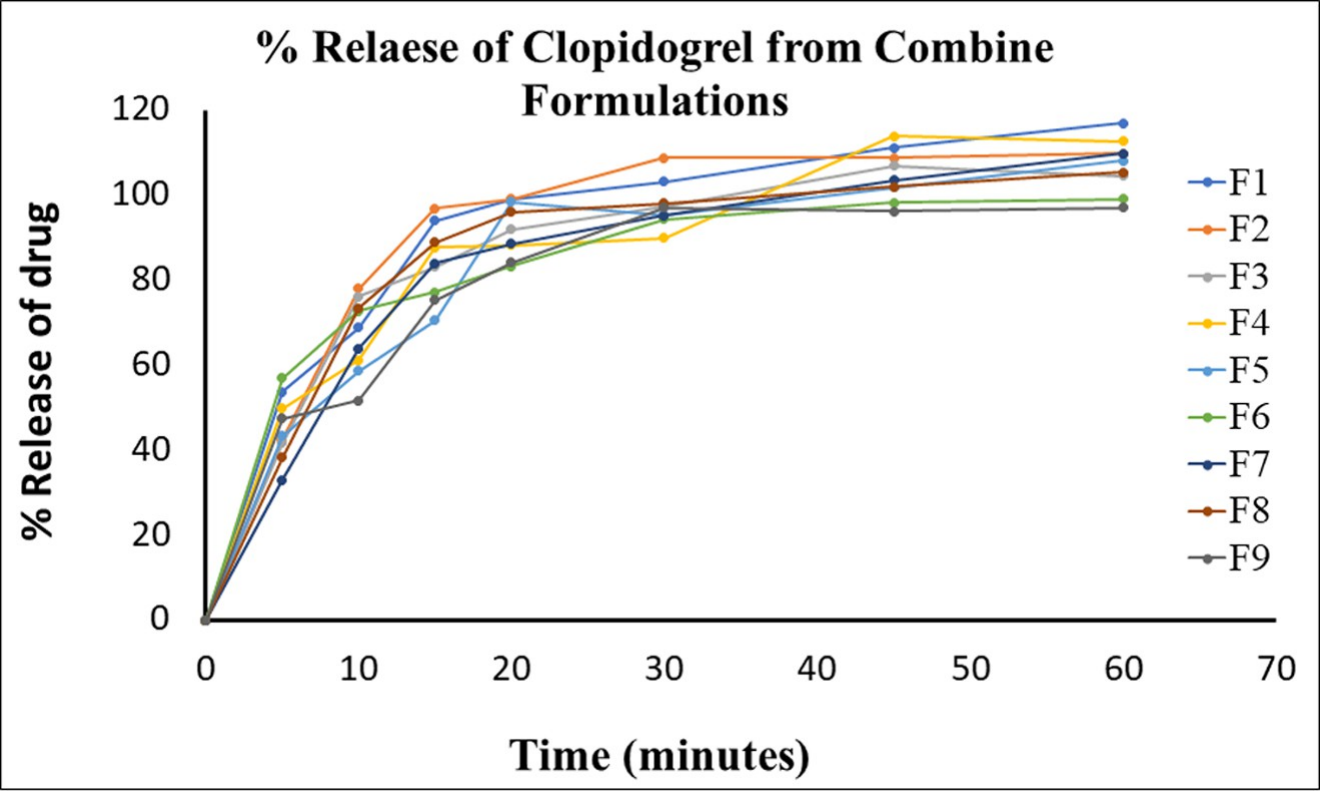
Graphical presentation of % release of clopidogrel from optimize combine formulation. Release profile of clopidogrel from different individual formulations (F1-F9).

**Fig 12:**
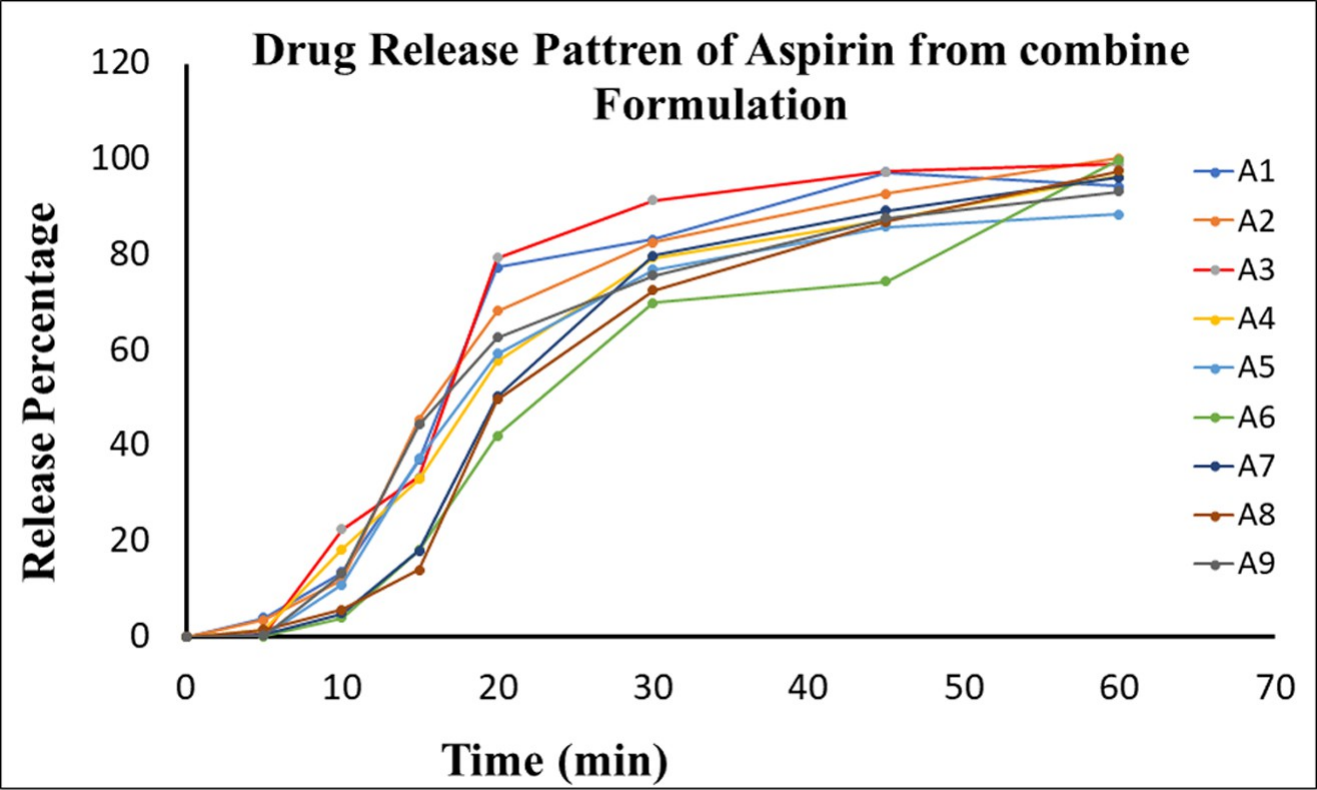
Graphical presentation of dissolution data of aspirin from optimize combine formulation. The drug release profile of aspirin from different individual formulations (A1-A9) when constructing a graph between release percentage versus time.

Dissolution is used as an in-vitro dynamic tool to check the content uniformity of drug (Q = 80% in 30 minutes) between different batches. The optimized batches (F1-F9) of A + CB were assessed for drug release from their dosage form by multi-point (5, 10, 15, 20, 30, 45 & 60 min) dissolution in 900 mL of dissolution medium (0.1N HCl). It was carried out for 6 tablets of each batch (F1 to F9) at rpm = 50 for 60 minutes. The average percentage release of clopidogrel after 15min (1–15 min of real time) was 70.42–96.82% with SD ± 8.71, where F5, F6 and F9 were only more than 80% in 20min. whereas aspirin was 69.88–91.49% in 15 min (between 15-30min of real time) with SD ± 6.41, all the batches released more than 80% in 20 min. The overall release contour of both the drugs after completion of 60 min was between 116.79–97.02% with SD ± 6.30 and 88.47–100.19% with SD ± 3.71, for clopidogrel and aspirin respectively. Therefore, the drug release pattern is one of the important characteristics of a drug delivery system which describes the drug dissolution profile inside the body (Figs 11 and 12).

To confirm the release order of the drug from formulated tablets (CPB-F8), the dissolution data was subjected to different kinetic models as presented in Table 7. The kinetics define the specific release pattern of drugs from their dosage form such as it may be dissolution dependent or diffusion dependent or the combination of these two. It also illustrates that the release might be concentration-dependent or independent or beyond these. Zero-order and Higuchi are used to verify the concentration-independent release whereas the first order helps to explain concentration-dependent release. Table 7 shows that all three marketed brands (STD_1CB_, STD_2CB_ & STD_3CB_) for clopidogrel failed to obey the zero-order and Higuchi Model, Likewise, CPB (F8) also failed to comply with these two models. The first-order, Hixson-Crowell and Weibull models described the drug release with r^2^ values of 0.9937, 0.9602 & 0.9963 respectively for clopidogrel that is identical to marketed brands, 0.9605–0.9983, 0.9602–0.9945 and 0.9727–0.9966 correspondingly. Similarly, F8 along with innovator and other generic products show the release of drugs with r^2^ value of 0.9763–0.9953 for first order; 0.9533–0.9896, except STD_2A_ for Higuchi; 0.9760–0.9878 for Hixson-Crowell and 0.9875–0.9999 for Weibull for aspirin in consolidation product (Table 7).

**Table 7:** Comparative data of dissolution models parameters of marketed brands & F8.

Parameter	For Clopidogrel				For Aspirin			
	STD_1CB_	STD_2CB_	STD_3CB_	F8_CB_	STD_1A_	STD_2A_	STD_3A_	F8_A_
**Zero Order–Model**								
**k**_**0**_	2.143	2.777	2.783	2.477	1.816	1.561	2.235	2.010
**R** ^**2**^	0.680	0.734	0.744	0.753	0.945	0.954	0.790	0.904
**First Order–Model**								
**k1**	0.050	0.408	0.154	0.125	0.031	0.029	0.132	0.080
**R** ^**2**^	0.996	0.960	0.998	0.993	0.976	0.994	0.995	0.980
**Higuchi Model**								
**kH**	13.237	19.565	18.954	16.902	13.803	15.531	0.009	10.051
**R** ^**2**^	0.783	0.822	0.836	0.841	0.953	0.872	0.976	0.989
**Hixson-Crowell Model**								
**kHC**	0.014	0.028	0.028	0.026	0.021	0.024	0.024	0.008
**R** ^**2**^	0.994	0.904	0.928	0.960	0.976	0.976	0.976	0.987
**Weibull Model**								
**Α**	23.449	164.801	164.801	6.996	32.861	11.657	1.185	2.192
**Β**	1.069	2.292	2.292	1.062	1.228	0.703	0.286	0.361
**Ti**	0.690	-1.805	-1.805	1.860	9.313	3.025	3.994	1.760
**R** ^**2**^	0.996	0.972	0.972	0.996	0.991	0.999	0.995	0.987

STD_CB_ = Marketed brands as standard to analyze Clopidogrel

STD_A_ = Marketed brands as standard to analyze aspirin

**F8**_**CB**_ **=** Check point batch to analyze clopidogrel

**F8**_**CBA**_ = Check point batch to analyze clopidogrel

In the case of aspirin, F8 along with STD_1A_, & STD_3A_ obeys the Higuchi & Hixson-Crowell model as well as first order and Weibull because the specific excipient in the formulation of aspirin tablets may control the release of drug by managing the concentration-dependent and independent atmosphere in tablet matrix. For example, pregelatinized starch is used as a filler-binder and works as a thickening and a gelling agent to make it suitable for zero-order release, first-order, the Higuchi model, even for Korsmeyer-Peppas model (0.9246–0.9863) also. Whereas Hixson-Crowell represents the dissolution rate of the drug as a function of time which means decrease in the surface area of a tablet with time. Weibull decides the best description when calculating the differences between the formulations (Table 7).

As per the model-dependent methods, the Weibull model demonstrated the best-fit model to the dissolution data between CPB and innovator along with generics brands. It appeared as high determination coefficient (R^2^ = 0.9727–0.9999) values. This is the model that described the dissolution profile in terms of shape (β represented shape parameter of the curve) and scale parameter (α). In the present study, the calculated β value was greater than ˃1 indicating the consistency of the material (Table 7). Weibull is considered a good model for the determination of differences among the various brands [51, 52].

**Fig 13:**
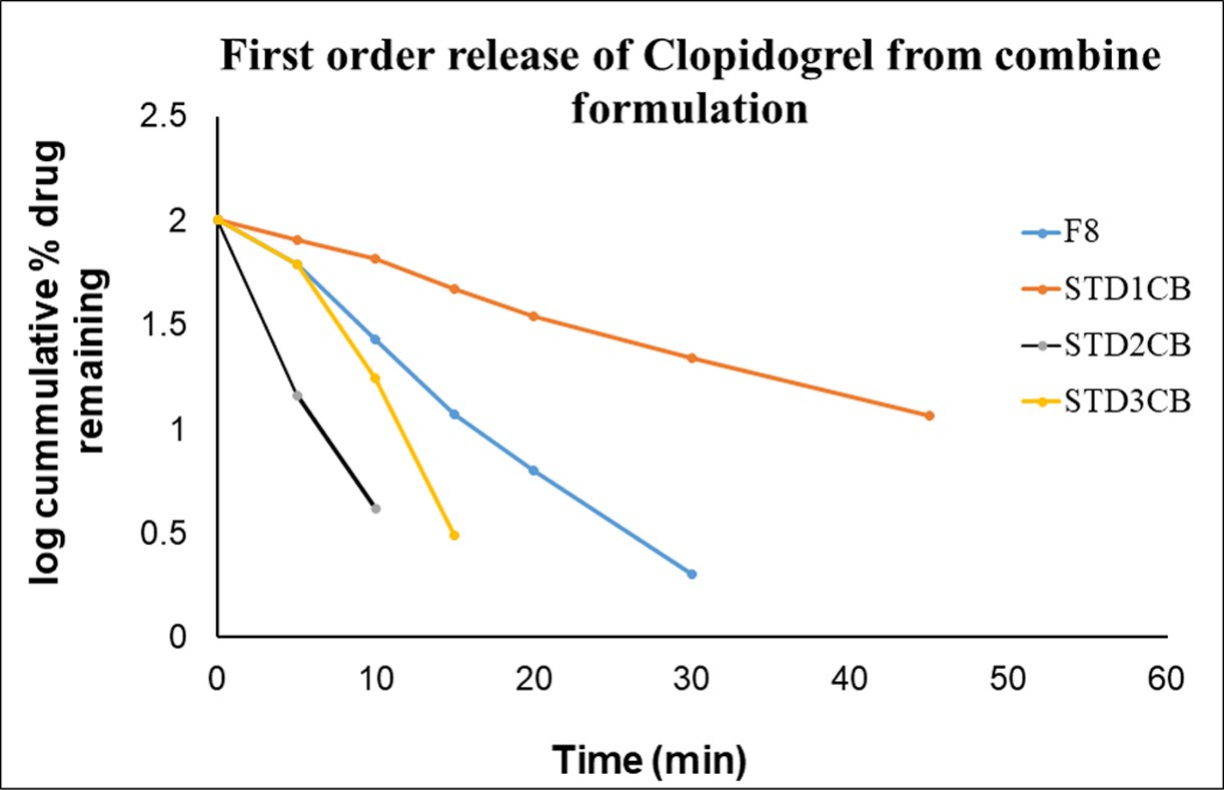
**Graphical presentation of First order release of clopidogrel from CPB and marketed brands.** F8- represents the release profile of Clopidogrel from the Check Point Batch (CPB); STD_1CB_- represents the release profile of Clopidogrel from one of the marketed brands of the medication; STD_2CB_- represents the release profile from 2^nd^ marketed brand and STD_3CB_- illustrates the rate of drug release from 3^rd^ marketed brand over time.

**Fig 14:**
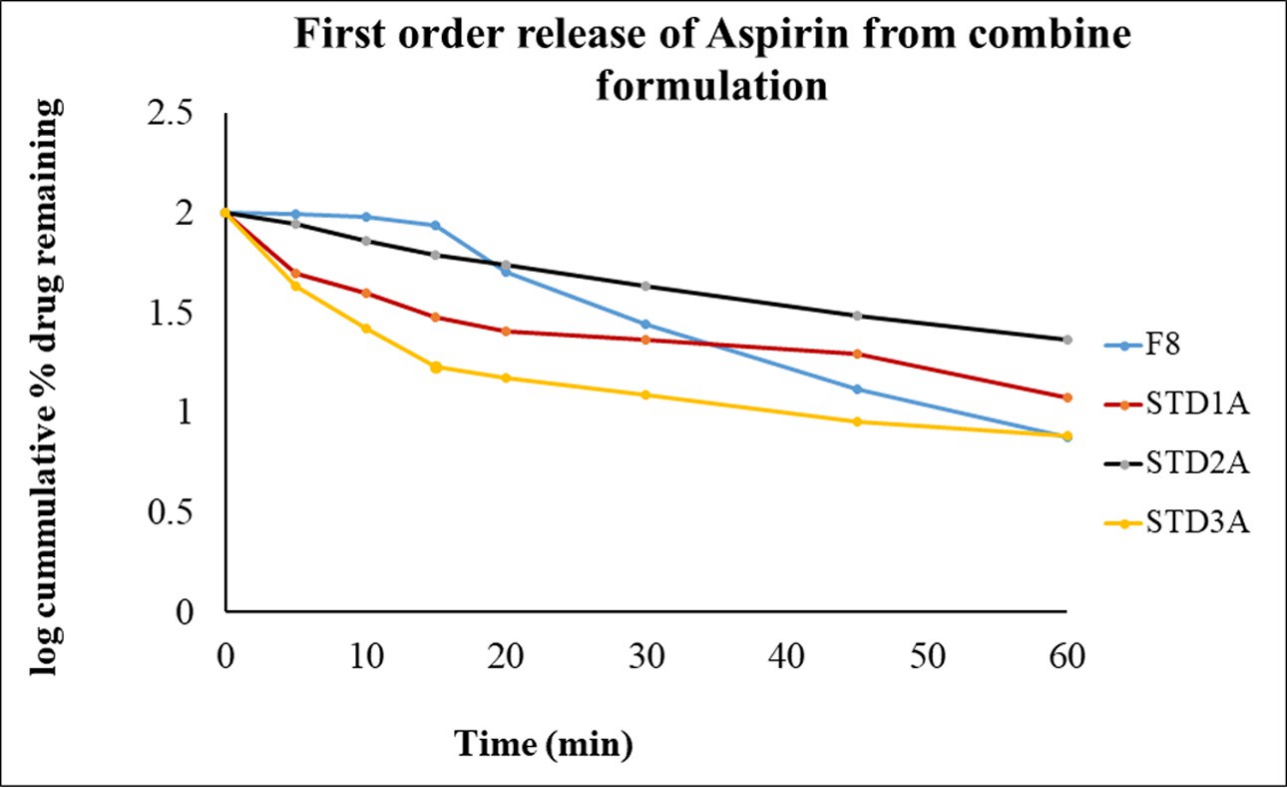
Graphical presentation of First-order release of aspirin from CPB and marketed brands. F8 represents the release profile of aspirin from the Check Point Batch (CPB); STD_1A_- represents the % release of aspirin from 1^st^ marketed brands; STD_2A_- illustrates the rate of drug release from 2^nd^ brand and STD_3A_- from the 3^rd^ marketed brand over time.

**Fig 15:**
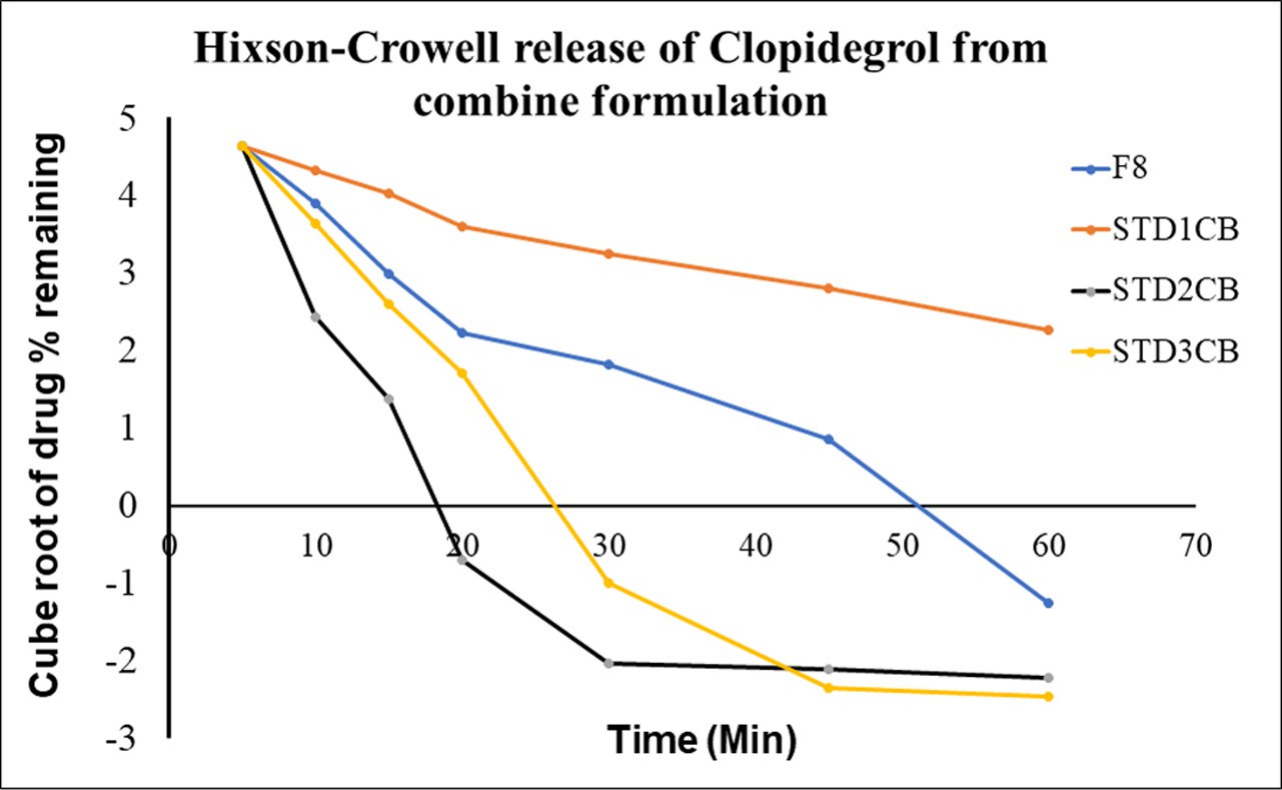
Graphical presentation of Hixson-Crowell release of clopidogrel from CPB and marketed brands. Dissolution profiles of check point batch (F8) for clopidogrel compared with dissolution profile of commercial tablets (STD_1CB_, STD_2CB_, STD_3CB_) after 60 minutes.

**Fig 16:**
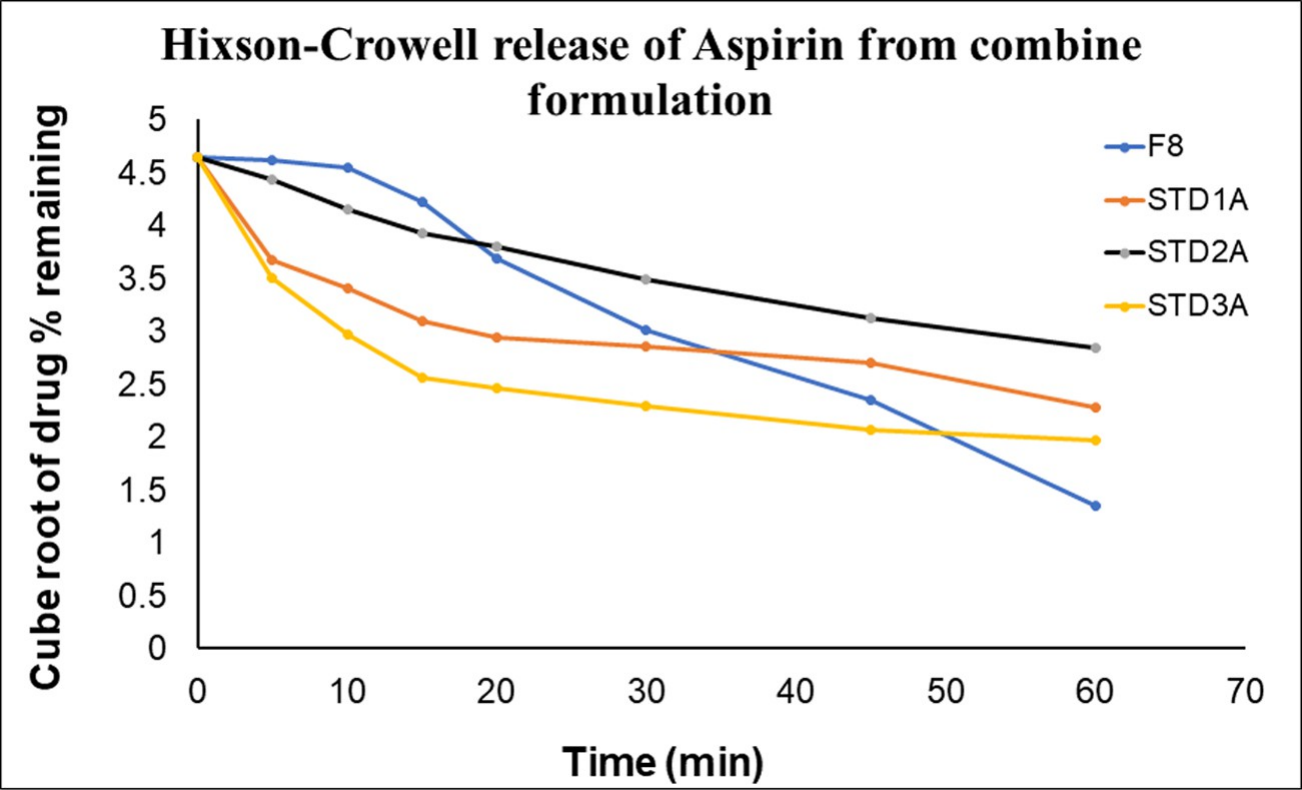
Graphical presentation of Hixson-Crowell release of aspirin from CPB and marketed brands. Dissolution profiles of checkpoint batch (F8) for aspirin compared with dissolution profile of commercial tablets (STD_1CB_, STD_2CB_, STD_3CB_) after 60 minutes.

The graphical presentation of First order (Figs 13 and 14) and Hixson-Crowell (Figs 15 and 16) indicated that the newly formulated tablets (CPB) are as good as the innovator and other generic tablets available in the market. The release of clopidogrel (Figs 13 and 15) and aspirin (Figs 14 and 16) is more than 80% within 20min that is identical as that of innovator (STD_1_).

In the present study, an attempt was made to prepare a combination tablet in such a way as to release both drugs rapidly (in 15 minutes) but in different time precincts. To validate and adjust the composition and proportion of the ingredients, a CPB based on the better contrast of independent variables was prepared and analyzed for their stability or compatibility of APIs with excipients at stress conditions (60 ± 2°C and 75 ± 5%) (Table 8). The assessment of drugs was carried out by stability indicating HPLC analytical method which was first developed then validated for intended purpose [18].

**Table 8:** Stress study (at 60 °C) of optimized prototype Formulation (CPB).

Test		Open			Amber		
Duration		0 day	7 days	15 days	0 day	7 days	15 days
**Weight Variation (mg)**		520.11±0.980	521.17± 0.014	519.28 ± 0.021	520.11±0.980	518.71 ± 0.027	521.34±0.023
**Hardness (Kp)**		4.75 ± 0.313	4.50 ± 0.375	5.125 ± 0.469	4.75 ± 0.313	5.50 ± 0.125	5.25 ± 0.188
**Friability (% w/w)**		0.33	0.31	0.37	0.33	0.36	0.41
Dissolution* (%)	**A**	100.01±0.83	107.76±0.31	106.83±0.67	100.01±0.83	102.1±0.56	108.51±0.43
	**CB**	101.81±0.22	101.22±0.89	102.61±1.27	101.81±0.22	102.9±1.16	102.01±0.94

No considerable deviation was observed in color, thickness, hardness and dissolution of the tablets. Based on the results, it was concluded that the excipients that were used into the formulation didn’t have any interaction among them and also, they have ability to protect characteristic of APIs. The stability outcomes of formulated tablets after 15 days of stress study indicated good compatibility between them (Table 8).

## Conclusion

It was concluded that the formulated tablets are efficacious, stable, and cost-effective. It is an innovative type of tablet where the release of both the drug from tablet was quick, without interrupting each other. It is different from conventional tablets as it comprises of two tablets with two different APIs, a small sized 75mg of aspirin (as an inner tablet) with 75 mg of clopidogrel used as a covering outer tablet. Most of the marketed tablets are bilayered or film-coated and release both drugs simultaneously that may sometime decrease the bioavailability of clopidogrel but does not affect its efficacy. Whereas some of the literature shows their synergistic affect may cause unusual bleeding, severe abdominal pain etc. The results of the study concluded that the intended combination formulation released the drugs instantaneously by the separate time interval to avoid any type of interaction among two APIs (S1–S3 Figs; S1 Table). Where, to improve the solubility of clopidogrel bisulphate as it is Class-II drug, polyethylene glycol 6000 was added by using simple mixing technique. On the other side, to hold aspirin, pregelatinize starch was used as gelling agent to control the release of drug.

These findings indicate that the study offers a new basis for dual therapy with clopidogrel and aspirin without making bilayer tablets. Further studies are required to confirm this proposition.

## Supporting information

S1 Fig Chromatographs of checkpoint batch (F8) dissolution at different time intervals. Graphical presentation of release pattern of Clopidogrel and Aspirin from combined formulated tablets.
(PDF)

S2 Fig Drug-drug compatibility study. Stability profile of the physical mixture (A + CB) at different intervals of time, i.e. 0, 5, 10 & 15 days.
(PDF)

S3 Fig Development and validation of HPLC analytical method. Summary of System suitability, linearity curve of Aspirin and Clopidogrel separately and in combined solution.
(XLSX)

S1 Table Analysis of pharmaceutical parameters of combined formulated tablets of A & CB. All the raw tables of weight variation, diameter, hardness, disintegration and drug release pattern of Aspirin and Clopidogrel from combined Formulation.
(XLSX)
