# Supplementary material for: Development and assessment of immediate-release tablets containing clopidogrel bisulphate & aspirin—strategy for optimizing the combination formulation
Source: PLoS One. 2024 May 23;19(5):e0303705. doi: 10.1371/journal.pone.0303705 (PMC11115251; doi:10.1371/journal.pone.0303705)

**S1_Fig: Chromatographs of check point batch (F8) dissolution at different time interval**

## Standard

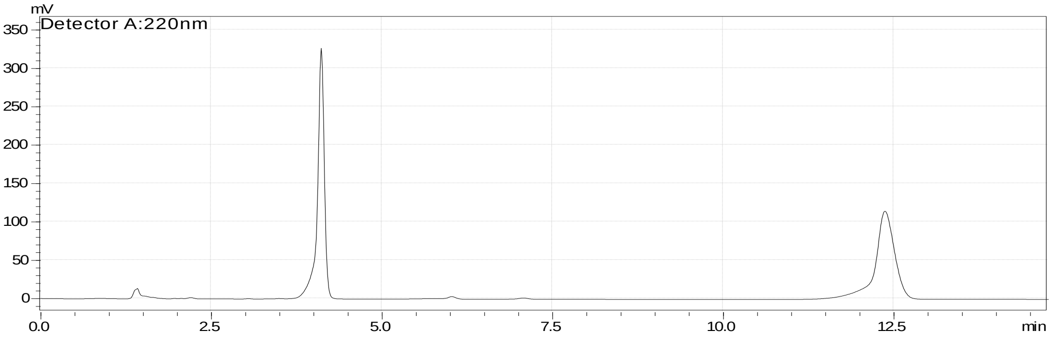


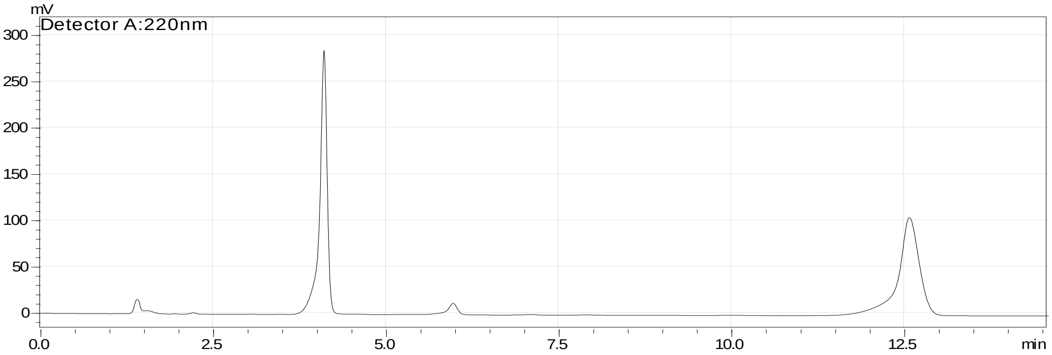


## F8: Sample 10 min

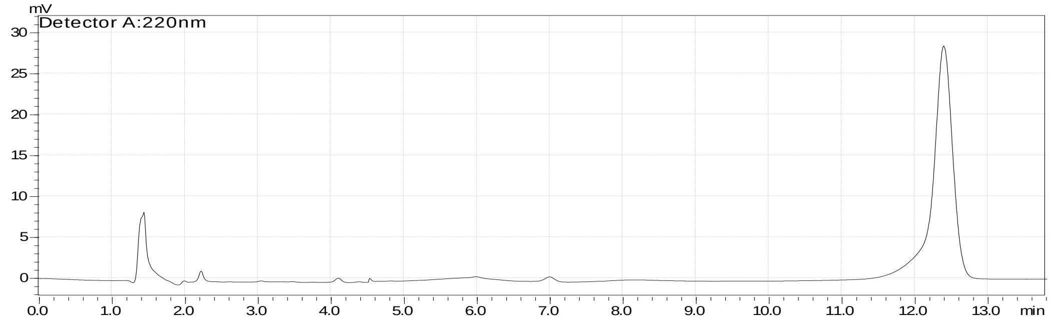


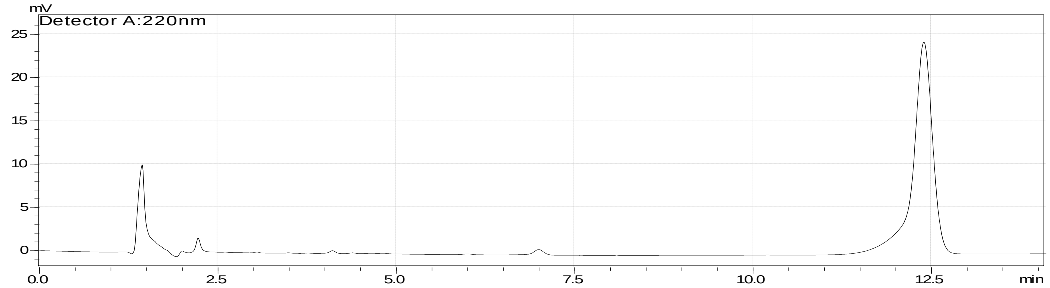


## Sample 15 min

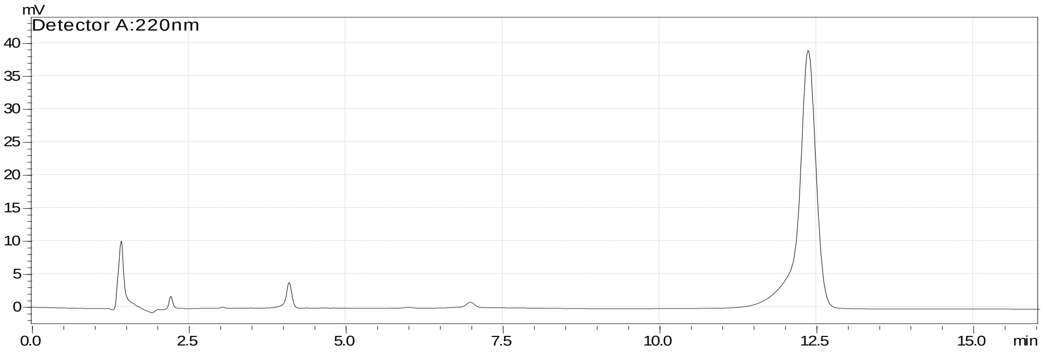


**Sample 20 min**

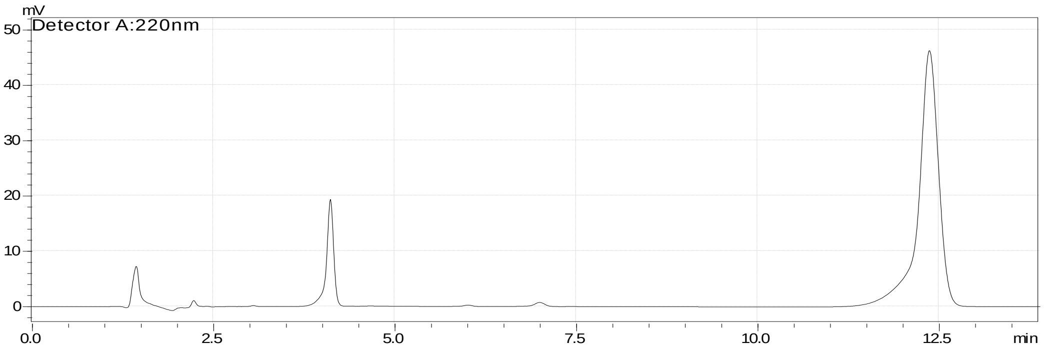


**Sample 30 min**

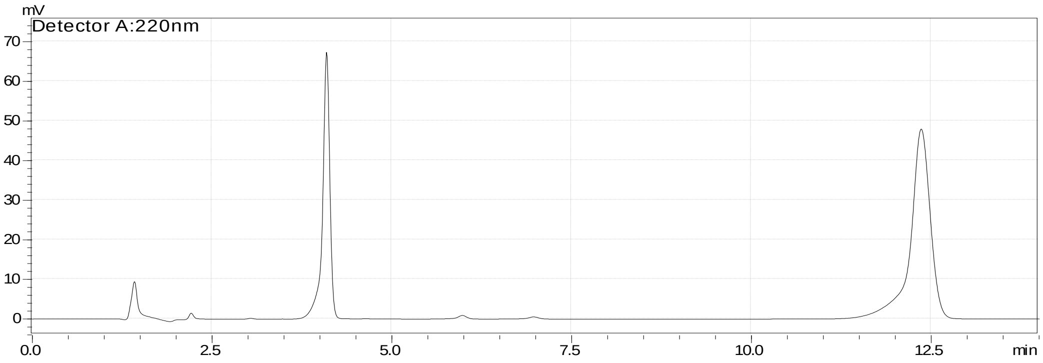


**Sample 45 min**

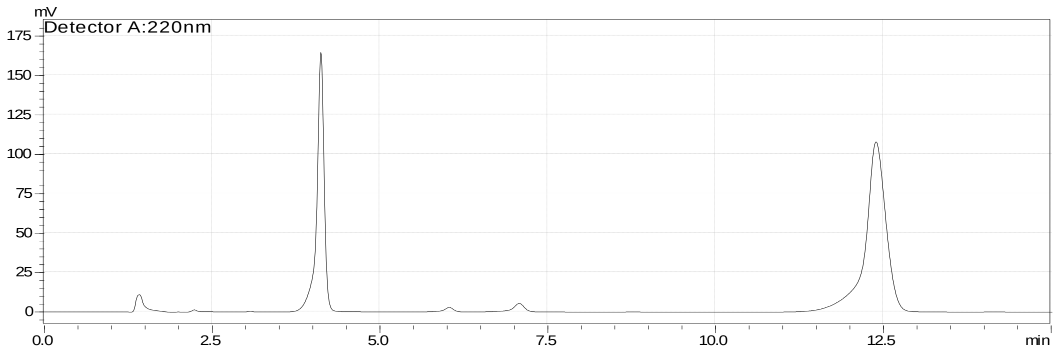

## Sample 60 min

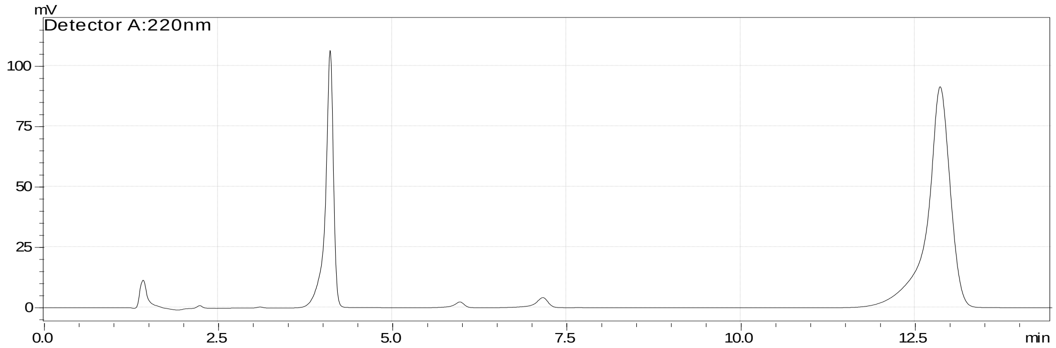


## Sample 90 min

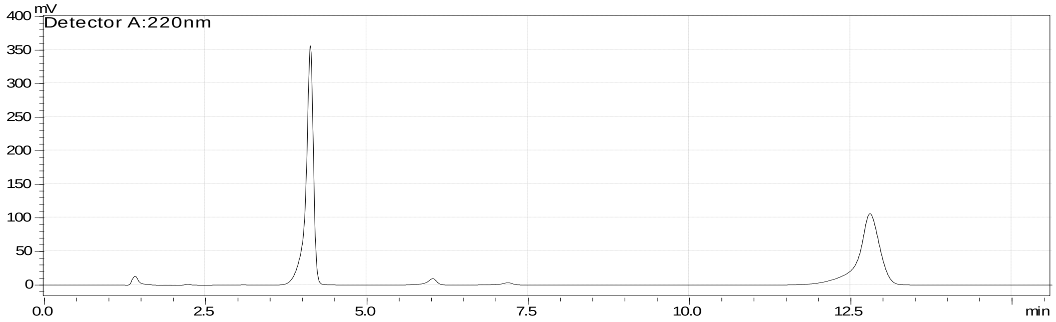


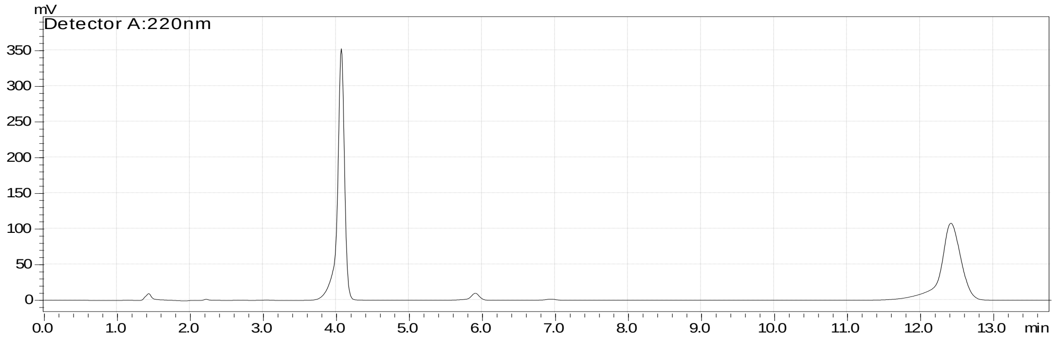

Supplement: S1 Fig — Graphical presentation of release pattern of Clopidogrel and Aspirin from combined formulated tablets. (PDF) [file pone.0303705.s001.pdf]
