# Supplementary material for: Development and assessment of immediate-release tablets containing clopidogrel bisulphate & aspirin—strategy for optimizing the combination formulation
Source: PLoS One. 2024 May 23;19(5):e0303705. doi: 10.1371/journal.pone.0303705 (PMC11115251; doi:10.1371/journal.pone.0303705)

S2 Fig. Drug-drug compatibility study

Day 1st Sample

60/80 microgram/ml

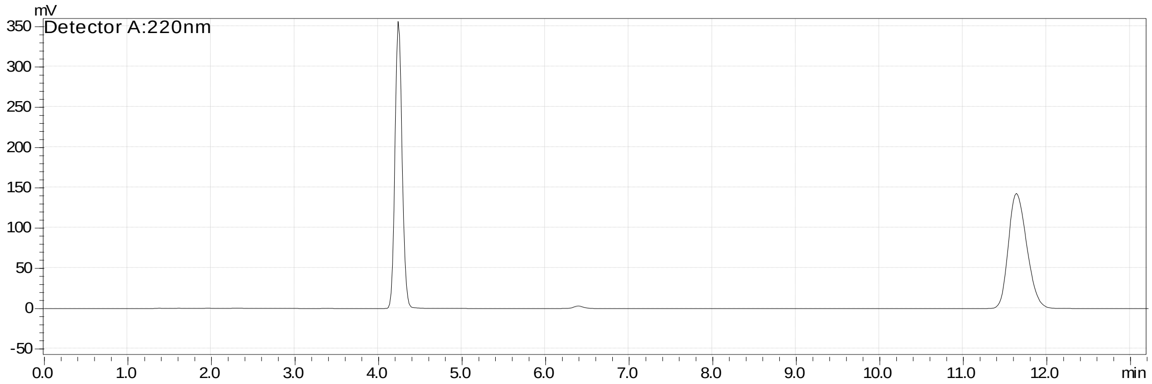

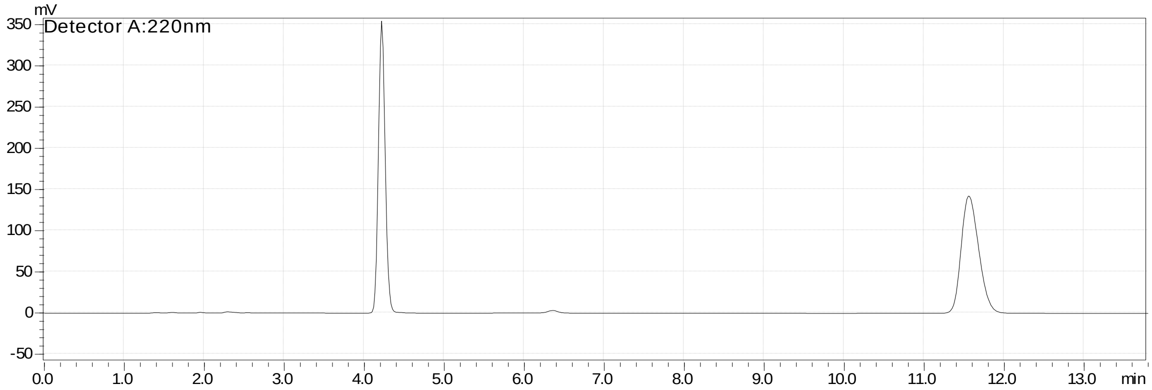

Day 5th (Sample-2)

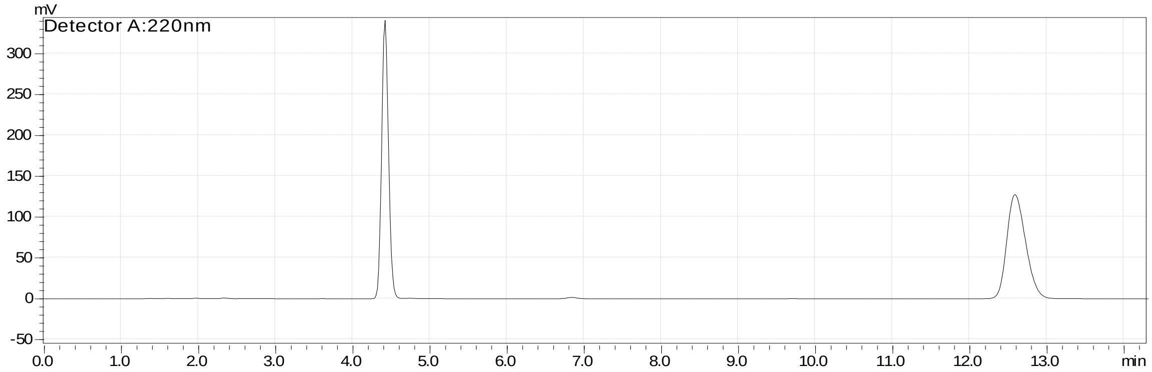

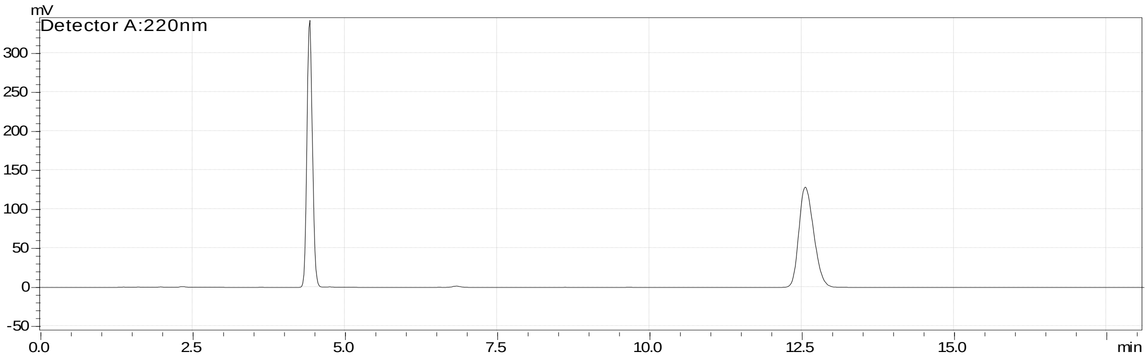

Day 5th (Sample-1)

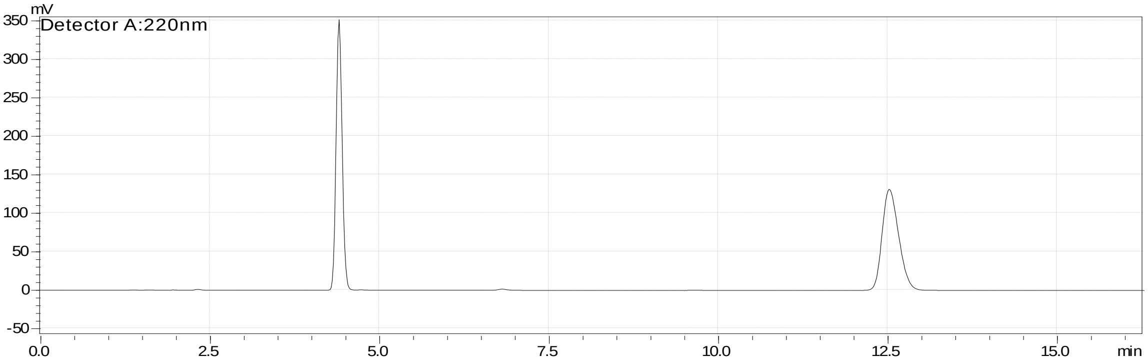

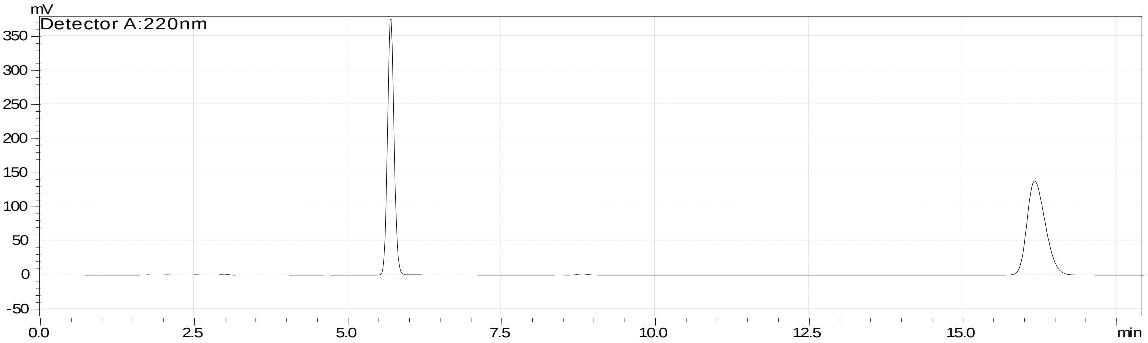

Day 10th (Sample-2)

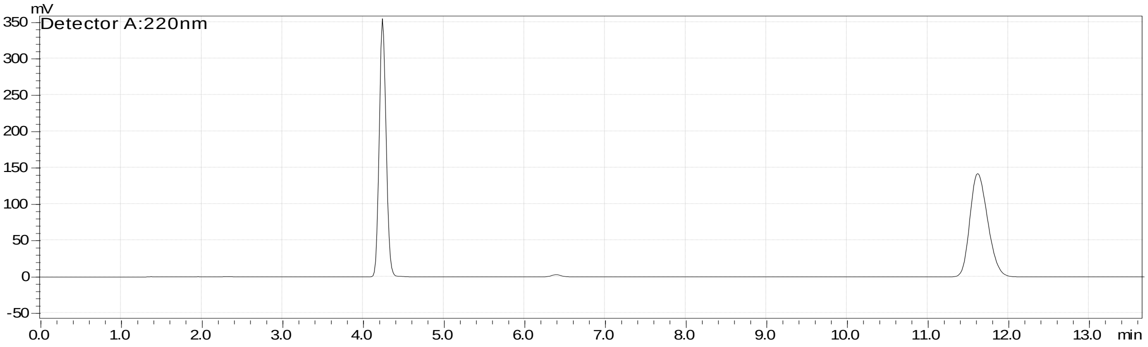

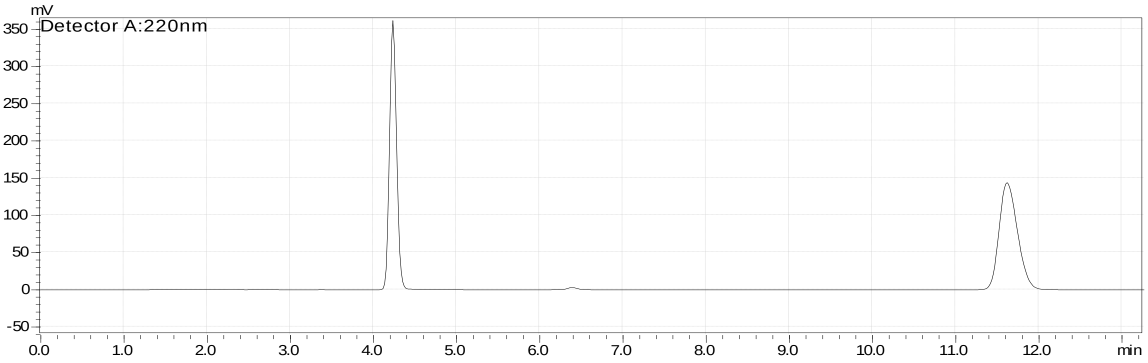

Day 10th (Sample-1)

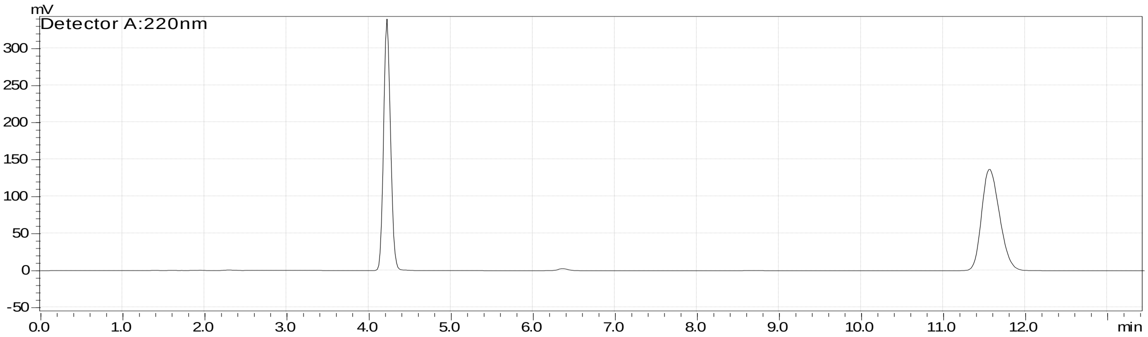

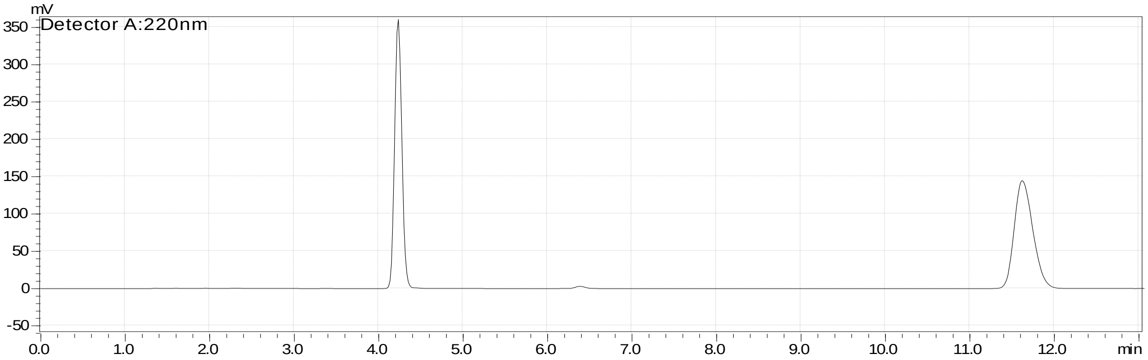

Day 10th (Sample-2)

15/20 microgram/ml

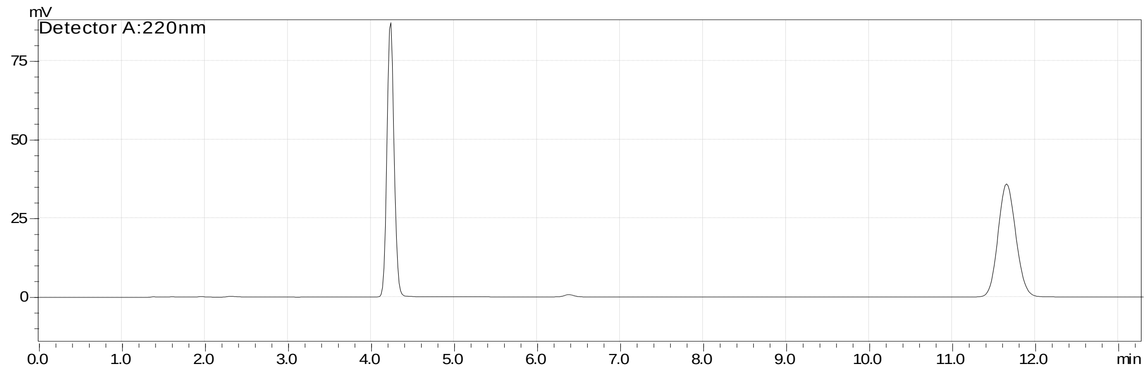

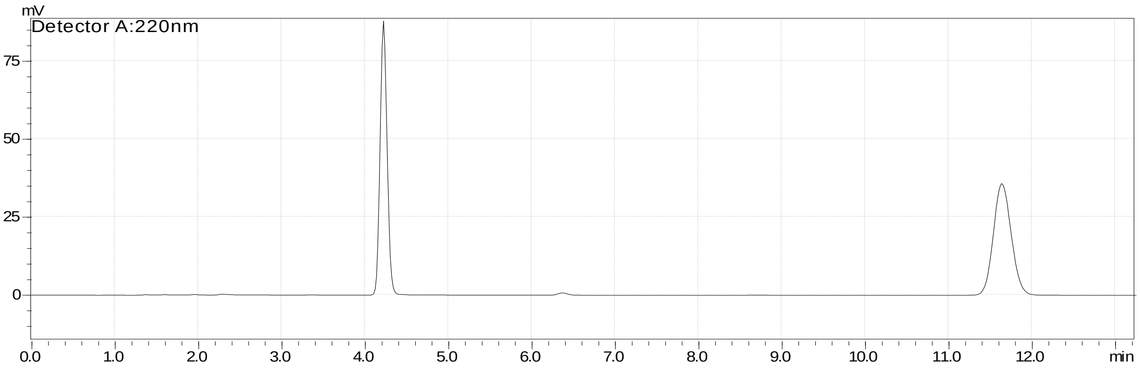

Day 10th (Sample-1)

15/20 microgram/ml

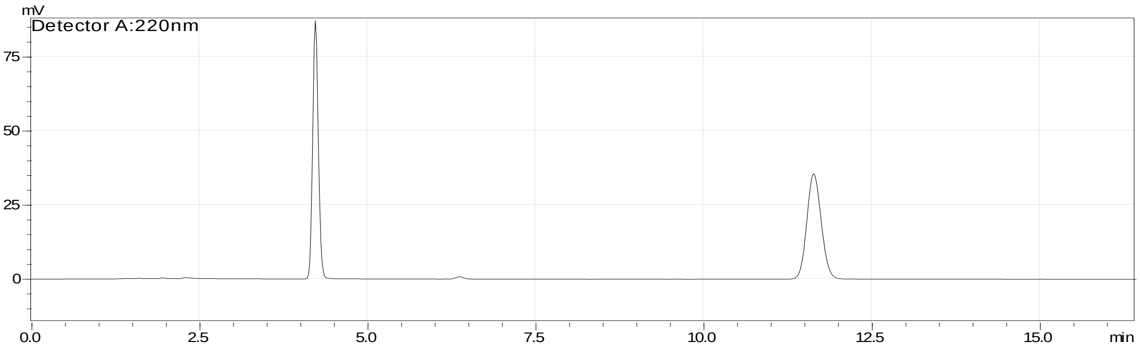

Day 15th (Sample-2)

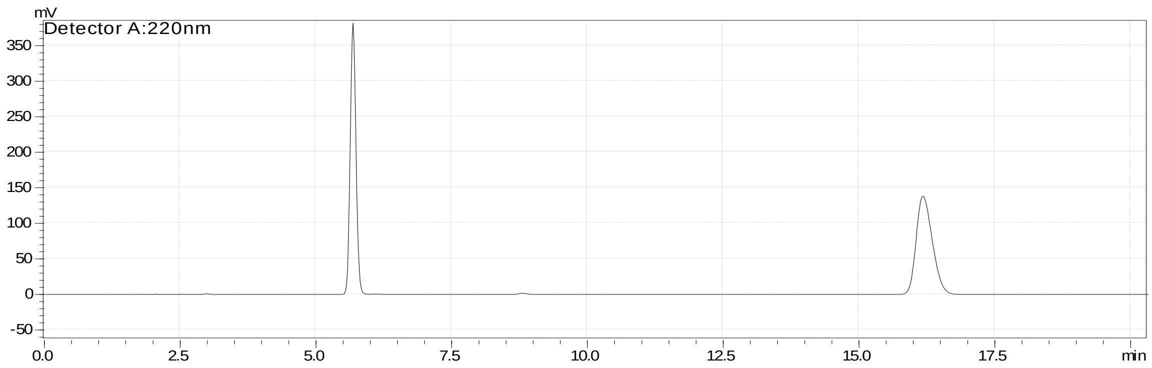

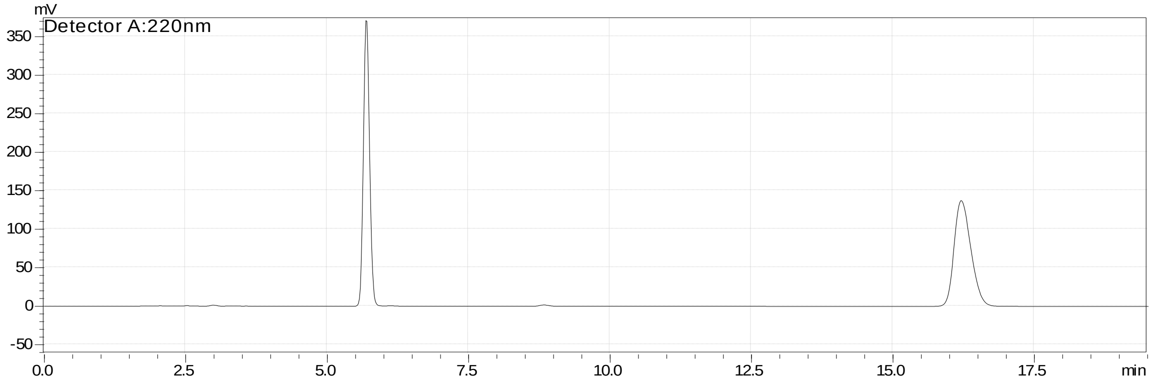

Supplement: S2 Fig — Stability profile of the physical mixture (A + CB) at different intervals of time, i.e. 0, 5, 10 & 15 days. (PDF) [file pone.0303705.s002.pdf]
